# Pelagic food web realignment supports resilient larvae of Southern Bluefin Tuna in a warming ocean

**DOI:** 10.1073/pnas.2601583123

**Published:** 2026-08-24

**Authors:** Michael R. Landry, Raúl Laiz-Carrión, Estrella Malca, Rasmus Swalethorp, Moira Décima, José M. Quintanilla, Ricardo Borrego-Santos, Claire H. Davies, Sven A. Kranz, Karen E. Selph, Michael R. Stukel, David Die, Lynnath E. Beckley, Barbara A. Muhling, Akihiro Shiroza, Lindsey E. Kim, Grace F. Cawley, Claudia Traboni, Kamran Walsh, Alejandro Jivanjee, Luke Matisons

**Affiliations:** ^a^ Scripps Institution of Oceanography, University of California San Diego, La Jolla, CA 92093; ^b^ Centro Oceanográfico de Málaga, Instituto Español de Oceanografía, Málaga 92002, Spain; ^c^ Rosenstiel School of Marine, Atmospheric, and Earth Science, University of Miami, Miami, FL 33149; ^d^ Departamento de Biología Animal, Facultad de Ciencias, University of Málaga, Málaga 92071, Spain; ^e^ Commonwealth Scientific and Industrial Research Organisation, Environment, Castray Esplanade, Hobart, TAS 7001, Australia; ^f^ Department of Biosciences, Rice University, Houston, TX 77251; ^g^ Department of Oceanography, University of Hawai’i at Manoa, Honolulu, HI 96822; ^h^ Earth, Ocean, and Atmospheric Science Department, Florida State University, Tallahassee, FL 32306; ^i^ Environmental and Conservation Sciences, Murdoch University, Murdoch, WA 6150, Australia; ^j^ Institute of Marine Sciences, University of California Santa Cruz, Santa Cruz, CA 95064; ^k^National Research Institute of Fisheries Science, Yokohama, Kanagawa 236-8648, Japan; ^l^ Integrative Marine Ecology, Stazione Zoologica Anton Dohrn, Naples 80121, Italy; ^m^ Coastal and Regional Oceanography Laboratory, University of New South Wales, Sydney, Sydney, NSW 2052, Australia

**Keywords:** larval tuna, appendicularian, larvacean, feeding, growth

## Abstract

Many of the oceans’ top predatory fishes at low to mid latitudes face an uncertain future as their vulnerable larvae deal with higher environmental temperature and reduced productivity associated with climate change. However, ocean ecosystem models that predict a general decline of zooplankton prey with warming and stratification hardly ever account for complexities in pelagic food webs that might make them resilient in providing food resources for larvae. We illustrate such complexities in comparing feeding interactions of larval Southern Bluefin Tuna (SBT) between two studies conducted 35 y apart in the same spawning region off northwest Australia. In the first (1987), SBT larvae fed and grew at low rates demonstrably limited by zooplankton prey. In the second (2022), feeding and growth were significantly higher despite substantially warmer conditions. The difference reflects a realignment in larval feeding preference from copepods to appendicularians, which allows a more direct and efficient energy transfer pathway from the microbially dominated food web base to higher-level consumers. These findings reset thinking on bluefin larvae feeding preferences, demonstrate high growth on an appendicularian diet up to 30 °C, and align with predictions of appendicularians being an environmentally selected zooplankton category in warming oligotrophic waters.

## Significance

Most trophic complexities are unconsidered in models that predict future ocean states, and critical interactions may also be poorly represented in historical knowledge. Comparing studies done 35 y apart, feeding and growth of Southern Bluefin Tuna larvae significantly improved due to a shift to a narrow diet of appendicularians, departing from the long-established trends of broad diet and crustacean prey for bluefin species. We make the case that appendicularians are the most efficient prey resource for bluefin larvae and will be environmentally selected as warming waters become less productive and dominated by small phytoplankton. As environmental conditions are altered, trophic adaptations within complex food webs can lead to substantially different species outcomes than expected from simple models and historical trends.

Southern Bluefin Tuna (SBT, *Thunnus maccoyii*) is one of three bluefin species (Atlantic–*T. thynnus*; Pacific–*T. orientalis*), the largest and widest-ranging tunas. All exploit rich feeding grounds in the temperate to subpolar regions of their respective oceans, and all are notable for undertaking long-distance migrations to reproduce in highly restricted, warm, and oligotrophic areas. Consistent with this pattern, SBT adults range broadly between 30 and 50°S throughout the Indian, eastern Atlantic, and western Pacific Oceans (1, 2), and their only known spawning region is a small tropical area between northwestern Australia and Indonesia (3–5). This area lies directly downstream of the Indonesian Throughflow (ITF), a globally significant but poorly studied region that is the only low-latitude connection between major oceans through which excess heat from the western Pacific enters the Indian Ocean (6, 7).

Over the past few decades, the long-term prospects for SBT have been concerning. Due to overfishing and stock decline, the International Union for Conservation of Nature (IUCN) placed SBT on its Critically Endangered list in 1996 (8), the only tuna to have been so designated at the full species level. Prior to that, in 1987, the last major study to have considered the distribution, feeding, and growth rates of SBT larvae in the ITF spawning region (9–12) painted a relatively bleak picture of the larval nutritional environment. Feeding incidence, the percentage of larvae with prey in their guts during daylight feeding hours, was very low (53%), and both prey consumption and growth rate were demonstrated to be limited by zooplankton prey (11, 12). In a 2015 global comparative analysis of larval tunas, mackerels, and billfish, SBT feeding conditions in the NW Australia spawning habit were noted to be unusually deficient, the lowest among all species examined (13).

In January–March 2022, the BLOOFINZ (Bluefin Larvae in Oligotrophic Ocean Foodwebs, Investigations of Nutrients to Zooplankton) Program conducted sampling and experimental studies in the SBT spawning area. With the objectives of assessing the determinants of larval habitat quality and evaluating potential vulnerabilities to climate change, BLOOFINZ combined traditional fisheries approaches for assessing feeding, growth, and survival of larvae with oceanographic analyses of biogeochemical limitations, productivity, and plankton food web ecology. While the full scope of results is presented elsewhere (14), here we highlight evidence and implications for substantially improved feeding and growth conditions that would not have been predicted by projecting the known historical relationships and behaviors of bluefin tuna larvae to the current ocean.

## Results and Discussion

### Contrasting Larval Habitat Conditions of 1987–2022.

**Fig. 1.**
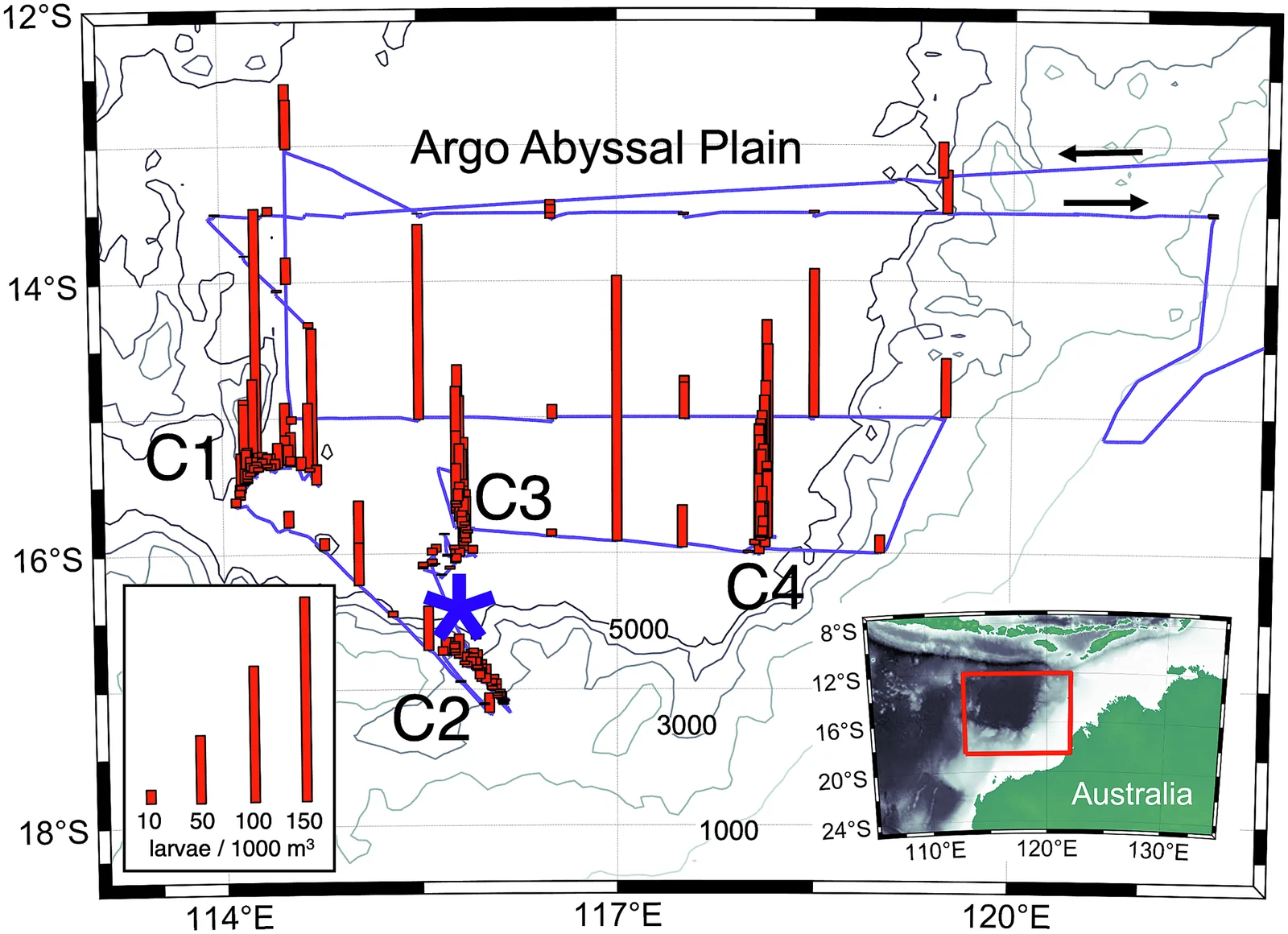
Abundances of SBT larvae (number/1,000 m^3^) along the BLOOFINZ cruise track. *Inset* shows study location in waters overlying the Argo Abyssal Plain (Argo Basin) off NW Australia. Arrows show directions of entry (26 January 2022) and departure (5 March 2022) from the study area. C1–C4 shows clusters of sampling results from multiday Lagrangian experiments following a satellite-tracked drifter. The blue asterisk is the center of SBT larval study area sampled in January–February 1987. Bathymetric contours are plotted at 1,000-m depth intervals.

SBT spawn in waters overlying the 5,000-m deep Argo Abyssal Plain (hereafter, Argo Basin). Our sampling of this region in 2022 revealed abundant and broadly distributed larvae in the southern basin, with densities declining in transects across the central basin and with shallowing bathymetry toward the Australian coastal margin (Fig. 1). The implication of this pattern, that spawning adults aggregate along the deep slope margin, is consistent with findings for larvae of Atlantic Bluefin Tuna in their offshore nursery habitat in the Gulf of Mexico, which backtrack to points of origin along the slope margin of NW Florida (15). The clusters of abundance estimates marked as C1–C4 in Fig. 1 correspond to high-frequency (typically ~4-h intervals) sampling during multiday Lagrangian experiments following a satellite-tracked drifter. Relative to the broader distribution sampled in 2022, the 1987 study was conducted in a smaller area (centered at the blue asterisk in Fig. 1) between our C2 and C3 Lagrangian experiments.

**Fig. 2.**
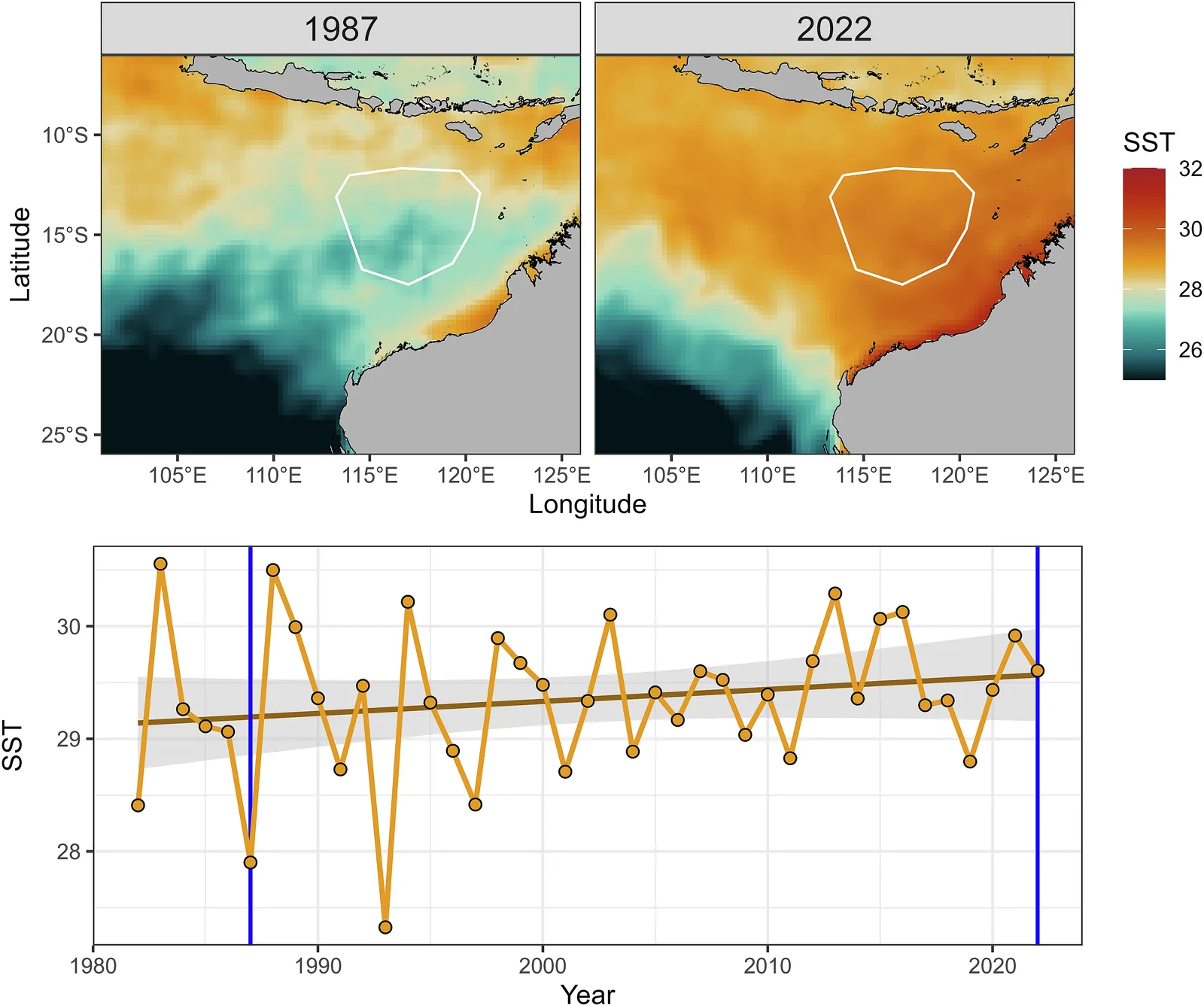
Temperature variability during the peak month of SBT spawning. (*Top*) Monthly mean distribution of SST in the eastern Indian Ocean study region in February 1987 and 2022. (*Bottom*) Time-series of mean February SST within the SBT spawning area (white polygon) from 1982 through 2022. A linear regression between year and SST is also shown, with 95% CI in gray shading. The two vertical blue lines highlight the 1987 and 2022 cruise years.

Sea surface temperature (SST) was substantially warmer during the peak spawning month of February in 2022 compared to 1987. Monthly mean SST distributions from satellite imagery showed no spatial overlap in any portion of the spawning area between years (Fig. 2, *Top*), and in situ temperature measurements from cruise sampling stations gave a mean difference of 2 °C (Table 1). In a four-decade SST time series for the month of February, however, 1987 stands out as anomalously cool (>1 °C below the trend line), while 2022 was more representative of the weakly increasing SST trend (+0.011 °C y^−1^; 95% CI = −0.01 to 0.03 °C y^−1^; Fig. 2, *Bottom*). Thus, much of the regional temperature difference is explained by interannual variability rather than general climate warming. Mean mixed-layer depth for 2022 was below the range reported for 1987, consistent with an expected warming effect on stratification (Table 1).

**Table 1.** Comparison of environmental, larval, and zooplankton variables from 1987–2022 studies in the Argo Basin spawning area

Variable	1987	2022
Sea-surface temp (°C)	27.46 ± 0.03	29.45 ± 0.06
Sea-surface salinity (psu)	34.81 ± 0.01	34.58 ± 0.01
Mixed layer depth (m)	16 to 22	14.1 ± 1.1
SBT larval density (larvae 1,000 m^−3^)	156 ± 37	33 ± 4
median (larvae 1,000 m^−3^)	12.1	14.0
SBT % of all tuna larvae (%)	82.1	89.5
SBT larval size (mm SL)	2.7 to 11.3	2.4 to 13.4
SBT feeding incidence (%)	52.7	94.0
SBT prey consumed (number gut^−1^)	0.85 ± 0.5	3.9 ± 0.18
SBT larval growth (mm d^−1^)	0.32 ± 0.01	0.39 ± 0.01
Copepods		
% in environment	87.1 ± 2.6	79.4 ± 1.3
% in diet	92.7	26.8 ± 4.4
Appendicularians		
% in environment	12.9 ± 2.6	10.6 ± 1.2
% in diet	0.66	58.9 ± 6.8

Uncertainties are ± SE of mean estimates.

In comparing the two studies, median estimates of larval SBT abundances were similar (12 vs. 14 larvae 1,000 m^−3^), as were the percent dominance of SBT larvae relative to all tuna species (82% vs. 90%) and the size range of SBT larvae collected (Table 1 and *SI Appendix*, Table S1). Average larval densities were higher in 1987 because the main emphasis of that study was on finding and repeat sampling of a high-density patch. In contrast, a high-density patch of recently spawned eggs (>82 eggs m^–3^ genetically confirmed to be SBT) was also found in 2022 but was not counted nor sampled repeatedly. Thus, both studies were characterized by comparable median larval densities with sporadic high-density patches where discrete adult spawning events had occurred.

In 2022, SBT larvae had a much higher feeding incidence during daylight feeding hours (94% vs. 53% of larvae with prey) and over four times higher prey consumed per larvae (3.9 vs. 0.9 prey per stomach) (Table 1). Growth rate estimates from standard length measurements and otolith daily increments were 22% higher in 2022 (0.39 vs. 0.32 mm d^−1^) (Table 1). This difference reduces the time required for a larva to grow from 3 to 10 mm by 4 d and, thus, would substantially improve survivorship through early life history assuming similar daily mortality losses to predators (i.e., not accounting for likely higher incidence of starvation mortality in 1987).

While compositions of zooplankton prey availability were relatively similar between studies for major groups like copepods (79% vs. 87%) and appendicularians (11% vs. 13%), their relative contributions to SBT larval diets differed greatly. The percentage of copepods in the larval diet declined by >threefold in 2022, from 93 to 27% (Table 1). Meanwhile, the contribution of appendicularians increased by >90 fold, from 0.7 to 59% of prey consumed. Overall, the dietary ratio of appendicularians to copepods consumed was more than 300 times higher in 2022.

### Appendicularians and Food Web Efficiency.

**Fig. 3.**
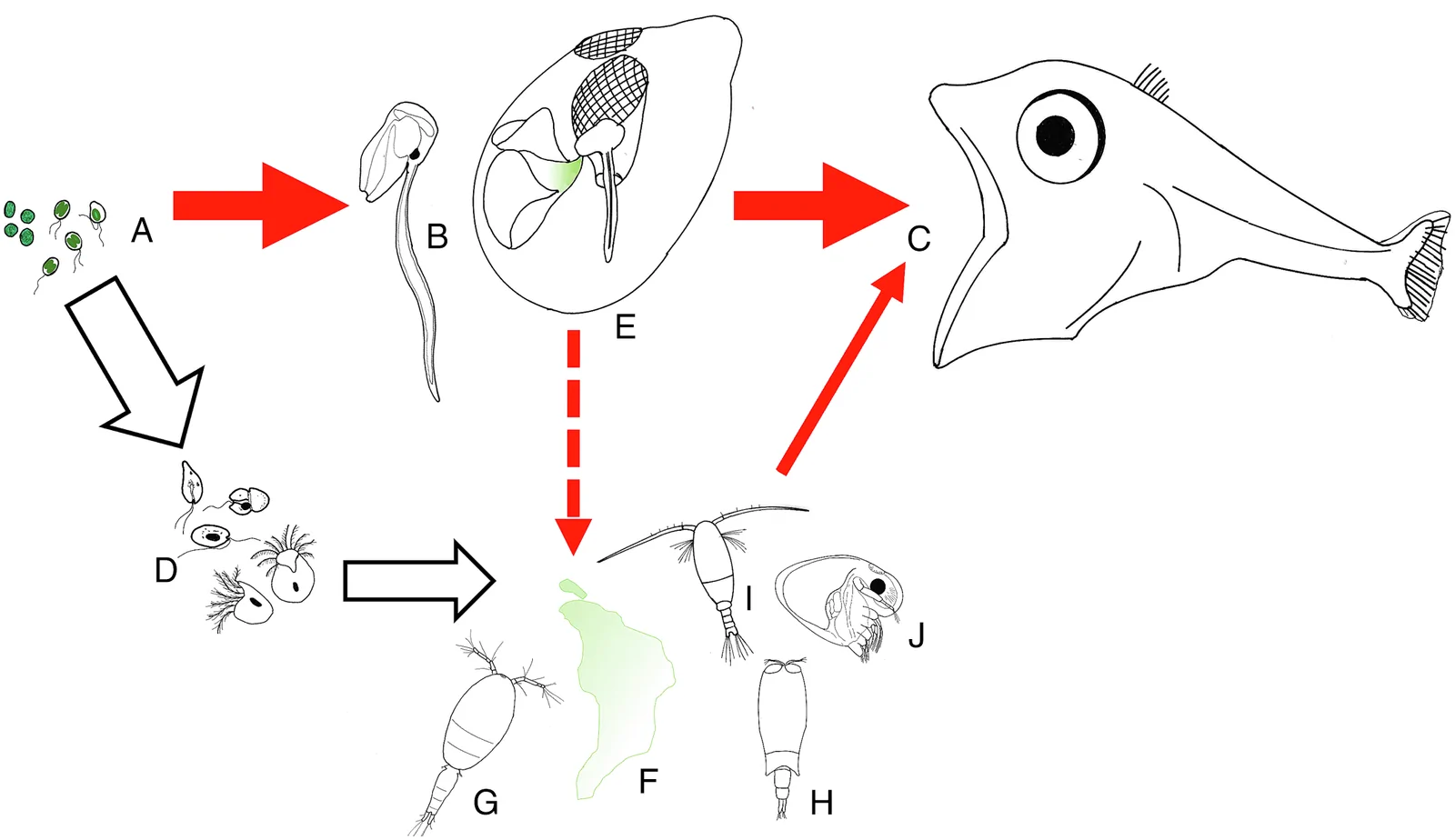
Appendicularian enhancement of food web transfer efficiency. Small picophytoplankton (*A*) are concentrated by appendicularians (*B*) using fine mucous-house filters, providing an efficient direct trophic transfer to SBT larvae (*C*) (heavy red arrows, 60% of diet biomass) that short circuits the longer pathway through intermediate levels of protistan consumers (*D*) in the microbial food web (large open arrows). Discarded appendicularian houses (*E* and *F*) with remnants of filter-concentrated phytoplankton also provide supplemental food to other zooplankton prey of larval tuna (copepods and cladocera) as large sinking particles (dashed red arrow), giving them access to small dominant phytoplankton that they would not be able to capture as individual food particles. Zooplankton pictured are cyclopoid copepods, *Oncaea* (*G*) and *Corycaeus* (*H*), calanoid copepod (*I*), and cladocera (*J*).

Appendicularians (also known as larvaceans) are small pelagic tunicates that live as individuals in secreted mucus houses with fine internal filters that allow them to concentrate bacterial-sized particles (16). In contrast to copepods of comparable size, they are uniquely equipped to access and feed efficiently on *Prochlorococcus* and other small phytoplankton (16–19) that dominate primary productivity in oligotrophic oceanic environments like the eastern Indian Ocean (20). Selective predation on appendicularians by tuna larvae therefore represents the most direct and efficient pathway of energy transfer from primary producers to larvae, short circuiting the more typical route through intermediate trophic steps of protistan consumers (21, 22) (Fig. 3).

Appendicularians also enhance food web efficiency by facilitating a more direct coupling of primary production transfer to other zooplankton. This is done by the frequent discarding of their mucus houses containing the remnants of filter-concentrated picophytoplankton, which then, as slowly sinking marine snow aggregates (23, 24), become a nutritionally rich food resource for the broader zooplankton community (Fig. 3). Cyclopoid copepods of the genus *Oncaea* are notable specialists that are strongly associated with attachment to, feeding upon, and degradation of discarded appendicularian houses (23, 25–27). Other documented associates of marine snow aggregates include Cladocera and Corycaeidae copepods (*Corycaeus*, *Farranula* spp.) (25), both among the most highly favored of tuna larvae prey (15, 28). Broader availability of particulate food resources for filter-feeding zooplankton should also occur as the houses degrade and fractionate in the euphotic zone (29). The presence of appendicularians thus benefits the entire community of zooplankton that serve as prey for larval tuna.

As a food resource themselves, appendicularians are nutritionally rich in proteins and fatty acids (30, 31) and have additional impressive qualities of relevance to ocean food web dynamics. Their rates of water filtration for food acquisition are an order-of-magnitude higher than those of comparably sized copepods (16, 32). They also greatly exceed co-occurring copepods in high individual growth rates, fast generation times, and high reproductive potential (33–36), and are more tolerant of higher temperature and increased seawater acidity (37–39). Based on potential as well as experimental and field observations, appendicularians are among the gelatinous forms that will gain an advantage with climate-driven changes of ocean ecosystems, and have been widely predicted to do so (37–42).

### Unexpected Dietary Flexibility.

Despite many attributes that make appendicularians a highly exploited food resource of numerous fish and invertebrate species (43), they are surprisingly poorly represented in historical dietary analyses of larval bluefin tunas (13, 28). This is exemplified by results from the 1987 study that showed very low feeding on appendicularians by SBT larvae (Table 1), even while skipjack tuna larvae (*Katsuwonus pelamis*) from the same net samples showed a high preference (50% of diet) for appendicularians (11). Global dietary comparisons have also highlighted the dichotomy between the broad larval diets of most *Thunnus* species with little reliance on appendicularians vs. the narrow specialization on appendicularians by other scombrid species that share the same habitats (13, 44, 45). The high SBT preference for appendicularians in our 2022 study, extending to all sampling sites and to all larval sizes and flexion stages [(46), *SI Appendix*, Table S2], thus represents not just the difference between two years in one system but a major departure from the established knowledge of many past studies.

Various hypotheses can be advanced to explain why the feeding preferences of SBT larvae shifted so dramatically between the 1987 and 2022 observations. The leading candidate would be a change in depth distribution of appendicularians. Supporting this idea, SBT larvae are known to feed closer to the sea surface than skipjack larvae (9). Thus, a deeper depth distribution of appendicularians in 1987 could explain why skipjack selected for appendicularians then, while SBT did not, without having to invoke innate difference in their abilities to perceive and capture prey (15). In 2022, near-surface abundances of appendicularians from a Continuous Plankton Recorder towed at 10 m depth were similar to the mean concentration from net tows in the upper 80 m. Unfortunately, there are no records or surviving samples to determine appendicularian densities at comparable shallow depths during 1987.

Altered composition of appendicularian species is another distinct hypothesis to explain the observed differences in feeding rates, as well as a mechanism that could contribute to study differences in depth distributions. While the diversity of appendicularian assemblages is poorly explored in most ocean systems, Sato et al. (42) reported 24 species from seven genera from a summertime sampling of a small but complex area of the western North Pacific. Species-specific differences in depth preferences, house sizes or robustness, and organism size, behaviors, or other factors (activity levels, temperature dependencies) that elicit or deter SBT predation are all possible reasons for a feeding shift to appendicularians in 2022. While appendicularian composition in 1987 is unknown, taxonomic analysis revealed that *Oikopleura longicauda* was the most common species in 2022, likely also accounting for most of the *Oikopleura* spp. too damaged for full identification. Together, these two categories averaged 66% of appendicularian abundance in the upper 30 m (*SI Appendix*, Table S3) and their trunk sizes (range = 0.06 to 1.00 mm; mean ± SD = 0.36 ± 0.13 mm) corresponded closely to the appendicularians found in SBT stomachs (0.12 to 0.87 mm; 0.36 ± 0.14 mm). In contrast, *Fritillaria* and *Megalocercus* spp. were larger on average than the appendicularians consumed (*SI Appendix*, Table S3), and *Fritillaria* spp. were much less prevalent where SBT were feeding (mean 12% of appendicularians in the upper 30 m vs. 39% of appendicularians in the upper 80 m). We can reasonably conclude from these results that the presence of abundant *O. longicauda* in near-surface waters would be a positive condition for bluefin larval feeding on appendicularians.

While we cannot resolve the exact reasons, among many, for dietary differences in two studies conducted decades apart, the 2022 findings are important for illustrating that radically different ecosystem outcomes can occur as complex pelagic food webs adapt to climate change. This result makes it clear that SBT larvae are not inherently disadvantaged in exploiting appendicularians as a food resource and could greatly benefit by doing so as conditions become more favorable. We can also infer from the fact that small and large SBT larvae from multiple sampling locations in 2022 displayed a common preference for appendicularians that some element of early feeding success (i.e., distributional overlap and the right prey characteristics) fostered a learned prey perception or hunting strategy that persisted throughout larval development (preflexion, flexion, and postflexion stages in *SI Appendix*, Table S2).

Due to the highly restrictive nature of bluefin tuna spawning areas in space and time, it is feasible to explore the range of conditions that determine the success of annual larval cohorts for these species over the typical duration of a research cruise, which is not possible for tropical tunas that spawn over wide portions of the oceans at many times of the year (47). Bluefin species, like SBT, thus provide convenient and accessible models for assessing how recruitment processes through early life stages are being impacted by environmental and ecological responses to changing climate. Nonetheless, given the high coherence of larval dietary preferences among bluefin species and among bluefins, *Thunnus* and billfish species in general (13), the lessons learned from larval bluefin studies also likely apply to the early life histories of many top ocean predators (28). One implication of the present results is that the diets of *Thunnus* and billfish larvae could become more similar to those of skipjack and other appendicularian-specialist scombrids if all exhibit high feeding preference for the same prey type, increasing competition among species occupying the same nursery habitats. Our observation of deeper depth distribution for *Fritillaria* spp. relative to *Oikopleura* spp. is a caveat in this regard as prey resources may still be strongly partitioned between surface-restricted bluefin species and other larvae, like skipjack, that feed deeper.

### Pelagic Food Web Complexities and Climate Change in Oligotrophic Larval Habitats.

Global earth system models project future warming and increased stratification of the oceans with relatively high confidence (48–53). While model agreement about primary production is lower (52), sharper declines are expected for zooplankton and their consumers, amplified by selection for smaller phytoplankton, longer food chains, and higher temperature-driven metabolic costs (54–59). One might readily conclude from such trends that environmental conditions within the restricted oligotrophic spawning grounds of bluefin tunas will become less conducive to successful recruitment as the waters warm, or that the main dimensions for adaptability will be distributional adjustments in space or time (earlier spawning). These assume that past correlated relationships with environmental variables, mainly temperature (47, 60–62), will remain fixed in the future.

Within physiological limitations, whether larvae can achieve higher growth potential with rising temperature depends on the adequacy and quality of the food resource environment (63–66). If interactions within complex pelagic food webs are altered as changing ocean conditions select for characteristics of zooplankton taxa, like appendicularians, that better fit the evolving physical realities (67), prey differences in distributional accessibility, catchability, and nutritional content can, in turn, produce different larval feeding and growth outcomes (68). What matters for bluefin species, in particular, is prey composition in near-surface waters during daytime relative to larval selectivity. In the current study, we document a high growth environment for SBT larvae in field observations up to 30 °C, where the warmest water conditions also had the highest larval feeding incidence and the largest proportion (≥70%) of appendicularians in their diet. Interpreted liberally, these findings could validate the widely predicted shift in ecosystem conditions favoring appendicularians (37–42, 67). More narrowly, they illustrate that even modest changes in this direction, imperceptible in the relative contributions of appendicularians to total zooplankton abundance between our two study years (Table 1), can have profound impacts on larval feeding and growth, enhancing survival through early development.

SBT remains overfished but has been slowly rebuilding stock at a 5% annual rate since its lowest level in 2009 (69). The species was upgraded from Critically Endangered to Endangered status in 2021 (70). The oceanic region off northwest Australia has also been recently identified as a global hotspot for many large pelagic fishes that utilize the habitat during austral spring/summer (71), presumably reflecting mutual benefits in retentive circulation, reduced predation, and nutritional environment that offset the disadvantages of increased interspecies competition (1, 71–73). Given climate change pressures, multispecies management goals would therefore be served by more frequent assessment of the habitat than every several decades. While observations 35 y apart cannot be taken to define a trend or to predict future success of the SBT species, the present results are significant in three important regards. They reset thinking on appendicularians not being highly selected prey of bluefin larvae. They align with predictions of appendicularians being efficient and environmentally favored consumers in warming oligotrophic waters. They demonstrate that selective feeding by SBT larvae on appendicularians can sustain high growth up to at least 30 °C. Future projections of climate change in the open oceans need to consider that improved efficiency in adaptive food webs could mitigate or at least greatly lessen the impacts on consumers like fish larvae through their critical early feeding stages.

## Methods

### Environmental Sampling.

The SBT spawning region was sampled between 14.9 to 16.7°S and 115.1 to 116.4°E by *FRV Solea* from 16 January to 2 February 1987 (74). *R/V Revelle* cruise RR2201 sampled the overlapping but larger area of 14.9 to 17.2°S, 114.1 to 119.5°E from 2 to 16 February 2022 (14). Both vessels were equipped for water-column profile sampling with a CTD (Conductivity-Temperature-Depth) system (NBIS Mk. IIIB in 1987; Seabird SBE 9plus in 2022), but the sensors were damaged during transit to the 1987 cruise and those records were unavailable. For that cruise, we used thermosalinograph measurements of water continuously pumped from 2-m depth and recorded at the start of each net tow (74) as the basis of comparison to CTD measurements of surface temperature and salinity from 2022. From each 2022 CTD hydrocast record at 1-m resolution, we computed mixed-layer depth (MLD) as the depth at which sigma-t density first exceeded the 0 to 5 m average by 0.1 kg m^−3^. For the 1987 study lacking CTD records, MLD estimates were reported as a range of values (9) from a Yeokal Submersible Data Logger with temperature and pressure sensors that was used to guide the depth sampling of each net tow, but the exact criterion for determining MLD was not given. For both cruises, samples for analyses of inorganic nutrients (nitrate, phosphate) were taken directly from CTD rosette bottles into acid-cleaned plastic containers, frozen at −20 °C, and analyzed in the laboratory by comparable colorimetric methods on semiautomated flowthrough systems (Tecator 5020 flow injection analyzer and Tecator 5023 spectrophotometer in 1987; Seal Analytical continuous-flow AutoAnalyzer in 2022). Sampling station measurements of surface mixed-layer temperature and salinity for the two cruises are included in *SI Appendix*, Table S1.

We also examined changes in sea surface temperature (SST) through time using monthly gap-free 0.25° NOAA Optimum Interpolation Sea Surface Temperature (OISST) Version 2.1 at https://psl.noaa.gov/data/obs/datadisplay/ (75). SST maps for February 1987 and February 2022 were created for the full area between 8 to 25°S, 105 to 125°E. To assess interannual variability along the temporal trend, an SST time series was determined based on the mean OISST product for the area overlying the Argo Basin for all Februarys between 1981 and 2022.

### Larval Tuna and Zooplankton Sampling.

In both 1987 and 2022 studies, larval tuna sampling involved net tows at random stations to locate patches as well as tows conducted during multiday experiments following a satellite-tracked buoy with a shallow drogue (20 and 15 m, respectively). In 1987, larval tuna were collected with a 70-cm bongo net system with paired 505-µm mesh nets towed obliquely through the mixed layer (average depth 42 ± 0.4 m) at 1.0 to 1.3 m s^−1^. In 2022, we used double oblique tows from the surface to a mean depth of 26 ± 0.6 m at 1.0 m s^−1^ with a 90-cm bongo net system with paired 505-µm mesh nets. Both net systems were outfitted with calibrated General Oceanic flowmeters, centered on each net mouth, to determine volume of water filtered to calculate larval abundance per 1,000 m^3^. The larvae collected in 1987 were preserved immediately in 95% ethyl alcohol (EtOH) on the cruise, and their standard lengths (SL, mm) were measured after preservation from the tip of the snout to the end of the notochord for preflexion larvae, or to the hypural crease for postflexion larvae (11). In 2022, the larvae collected on one side of the bongo nets were generally sorted to species and their standard lengths (SL, mm) measured fresh on shipboard before preserving in EtOH. Many of the same specimens were remeasured later in the laboratory to establish a relationship for the preservation shrinkage effect. Both collections were handled similarly with respect to replacing the original preservative with fresh EtOH after the first 24 h. Here, we corrected preserved SL from 1987 to fresh SL using the relationship from 2022; SL_fresh_ = 1.204 × SL_EtOH_ – 0.522. Station estimates of larval tuna abundances are in *SI Appendix*, Table S1.

For both 1987 and 2022 studies, the zooplankton community of potential larval tuna prey was sampled with a Heron drop-net with 60-cm mouth and 100-µm mesh (76), the reference standard for Australia’s Integrated Marine Observing System (77) due to its operational simplicity. The weighted net has no preceding bridle, is dropped mouth first from the surface while the ship is stationary, and is cinched closed at the bottom of the cast before retrieval. The Heron net drops were done to a mean depth of 60 m in 1987 and to 80 ± 2 m depth in 2022, and the samples were preserved with 5% and 10% formaldehyde, respectively. The 1987 samples were divided with a Folsom splitter, with one half retained for biomass measurement and the other for estimates of prey numbers. The 2022 net drops were done in duplicate immediately after one another, with one drop used for biomass and the other for prey abundance.

### Zooplankton Biomass and Abundance.

Biomass samples from 1987 were size fractioned through mesh sizes of 1,000, 333, and 100 µm, rinsed with fresh water to remove preservative and salt, and each fraction was dried on preweighed filter paper at 70 °C for 24 h before reweighing for the dry mass difference (78). In 2022, the total sample of the biomass tows were reweighed for dry mass after being rinsed with fresh water on a 70-µm mesh sieve, transferred to preweighed petri dishes, and dried for 24 h at 60 °C. We combined the 1987 dry-weight size fractions to compare with the total 2022 sample dry weights, both of which were corrected for the volumes of water filtered.

In both studies, samples for zooplankton compositional analyses were subsampled volumetrically with a Stempel pipette and enumerated microscopically. However, taxonomic details are only available for the 2022 samples (139 zooplankton taxa, including >100 copepod categories to genus or species), while the 1987 results were reported in broad prey groupings: copepod nauplii, cyclopoid copepodites, calanoid copepodites, appendicularians, and cladocerans. For comparisons, the taxon-specific volume-corrected counts from 2022 were combined into the same broad categories as those from 1987.

Species composition of appendicularian prey are unknown for 1987. For 2022, the assemblage available to SBT larvae was determined from composite subsamples of noontime net tows (200-µm mesh) in the upper 30 m for each of the Lagrangian experiments (C1–C4; Fig. 1). All appendicularians were picked from the subsamples, measured for trunk and tail lengths, and identified to species (or genus level when specimen condition precluded full identification). These data are presented in *SI Appendix*, Table S3 as summaries of species’ lengths and mean percent compositions, followed by results for each specimen analyzed.

### Larval Tuna Feeding and Growth.

In both studies, feeding indices were determined from microscopical analyses of the stomach contents of randomly selected larval tuna specimens from daytime net collections in comparison to the composition of the zooplankton prey community [(11, 79); *SI Appendix*, Table S2]. “Feeding incidence” is the percentage of stomachs containing at least one food item. “Prey number” is the mean number of prey per larva. “Percent diet” is the mean percent of a specific prey type in the larval diet.

Larval growth rates were determined from analyses of the sagittal otoliths extracted from the cranial cavity of selected specimens (10, 80). The otoliths were fixed with one drop of mounting medium and examined and digitized (H.E.C. “Video coordinate digitizer” at 920 to 2,360× in 1987; Leica CTR6 LED microscope at 1,000× with Leica X LAS X 2.0 software in 2022). Daily growth rings were counted along the longest axis of the otolith core to the edge (81). Since most otoliths do not have a clear hatch mark, a minimum distance of 7 μm from the central core was designated as the starting point (10, 61), and increments were added until reaching the first clearly observed increment. A terminal increment was added if there was space before the outer radius (82). Larval age refers to days post hatch (dph).

## Supplementary Material

Appendix 01 (PDF)

## Data Availability

Environmental data and larval tuna abundances for 1987 and 2022 studies are tabulated in *SI Appendix*, Table S1. Prey ingested by SBT larvae in 2022 are tabulated in *SI Appendix*, Table S2. Data on sizes and species of appendicularians in 2022 are tabulated in *SI Appendix*, Table S3. All other data products in Table 1 from the 1987 study were extracted from the original published papers (10–12, 74).
