# Supplementary material for: Pelagic food web realignment supports resilient larvae of Southern Bluefin Tuna in a warming ocean
Source: Proc Natl Acad Sci U S A. 2026 Aug 24;123(35):e2601583123. doi: 10.1073/pnas.2601583123 (PMC13535185; doi:10.1073/pnas.2601583123)
Supplement: Supplementary file 1 [file pnas.2601583123.sapp.pdf]

**SUPPORTING INFORMATION**

# Pelagic food web realignment supports resilient larvae of Southern Bluefin Tuna in a warming ocean

Michael R. Landry[a*], Raúl Laiz-Carrión[b], Estrella Malca[c], Rasmus Swalethorp[a], Moira Décima[a], José M. Quintanilla[b], Ricardo Borrego-Santos[b,d], Claire H. Davies[e], Sven A. Kranz[f], Karen E. Selph[g], Michael R. Stukel[h], David Die[c], Lynnath E. Beckley[i], Barbara A. Muhling[j], Akihiro Shiroza[k], Lindsey E. Kim[a], Grace F. Cawley[a], Claudia Traboni[l], Kamran Walsh[a], Alejandro Jivanjee[c], Luke Matisons[i,m]

[a] Scripps Institution of Oceanography, University of California, San Diego, La Jolla, CA, USA
[b] Centro Oceanográfico de Málaga, Instituto Español de Oceanografía (IEO-CSIC), Spain
[c] Rosenstiel School of Marine, Atmospheric, and Earth Science, University of Miami, Miami, Florida, 33149, USA
[d] Departamento de Biología Animal, Facultad de Ciencias, Univ. Málaga, Spain
[e] CSIRO Environment, Castray Esplanade, Hobart, Tasmania, Australia
[f] Department of Biosciences, Rice University, Houston, TX, USA
[g] Department of Oceanography, University of Hawai'i at Manoa, Honolulu, HI, USA
[h] Earth, Ocean, and Atmospheric Science Dept., Florida State University, Tallahassee, FL, USA
[i] Environmental and Conservation Sciences, Murdoch Univ., Murdoch, WA, Australia
[j] Institute of Marine Sciences, University of California, Santa Cruz, Santa Cruz, CA, USA
[k] National Research Institute of Fisheries Science, 2-12-4 Fukuura, Kanazawa, Yokohama, Kanagawa, 236-8648, Japan
[l] Integrative Marine Ecology, Stazione Zoologica Anton Dohrn, Naples, 80121, Italy
[m] Coastal and Regional Oceanography Laboratory, UNSW Sydney, 2033, NSW, Australia

*Corresponding author*

***Abstract***

Many of the oceans' top predatory fishes at low to mid latitudes face an uncertain future as their vulnerable larvae deal with higher environmental temperature and reduced productivity associated with climate change. However, ocean ecosystem models that predict a general decline of zooplankton prey with warming and stratification hardly ever account for complexities in pelagic food webs that might make them resilient in providing food resources for larvae. We illustrate such complexities in comparing feeding interactions of larval Southern Bluefin tuna (SBT) between two studies conducted 35 years apart in the same spawning region off northwest Australia. In the first (1987), SBT larvae fed and grew at low rates demonstrably limited by zooplankton prey. In the second (2022), feeding and growth were significantly higher despite substantially warmer conditions. The difference reflects a realignment in larval feeding preference from copepods to appendicularians, which allows a more direct and efficient energy transfer pathway from the microbially dominated food web base to higher-level consumers. These findings reset thinking on bluefin larvae feeding preferences, demonstrate high growth on an appendicularian diet up to 30°C, and align with predictions of appendicularians being an environmentally selected zooplankton category in warming oligotrophic waters.

**Table S1.** Abundances of tuna larvae (number/ 1000 $m^3$) and environmental conditions during 1987 and 2022 studies in the Argo Basin. Vol ($m^3$) = water volume sampled by net tow. SBT = Southern Bluefin (*Thunnus maccroyii*); ALB = Albacore (*T. alalunga*); YFT = Yellowfin (*T. albacares*); BET = Bigeye (*T. obesus*); SKJ = Skipjack (*Katsuwonus pelamis*); Tot Tuna = All tuna larvae; Other = Other larval fishes. MLD = Mixed Layer Depth; Temp = temperature; Sal - Salinity.

| | | | | | | | LARVAE /1000 $m^3$ | | | | | | | | | | |
|---|---|---|---|---|---|---|---|---|---|---|---|---|---|---|---|---|---|
| **Date** | **Stn/Tow #** | **Time GMT** | **Latitude** | **Longitude** | **Depth (m)** | **Vol ($m^3$)** | **SBT** | **ALB** | **YFT** | **ALB/YFT** | **BET** | **SKJ** | **Tot Tuna** | **Other** | **MLD (m)** | **Temp (°C)** | **Sal (psu)** |
| 16-Jan-1987 | 128A | 1321 | -16.545 | 115.344 | 35 | 593 | 0 | 0 | 0 | 0 | 0 | 0 | 0 | 27 | | 28.58 | 34.85 |
| 16-Jan-1987 | 128B | 1321 | -16.545 | 115.344 | 35 | 593 | 0 | 0 | 0 | 0 | 0 | 0 | 0 | 19 | | 28.58 | 34.85 |
| 16-Jan-1987 | 132A | 1724 | -16.546 | 115.342 | 34 | 806 | 0 | 1 | 0 | 0 | 0 | 4 | 5 | 46 | | 28.50 | 34.86 |
| 16-Jan-1987 | 1328 | 1724 | -16.546 | 115.342 | 34 | 806 | 5 | 0 | 0 | 0 | 0 | 5 | 12 | 42 | | 28.50 | 34.86 |
| 17-Jan-1987 | 139A | 0235 | -16.440 | 115.555 | 37 | 617 | 13 | 2 | 0 | 2 | 0 | 3 | 23 | 164 | | 28.15 | 34.80 |
| 21-Jan-1987 | 139B | 0235 | -16.440 | 115.555 | 37 | 617 | 6 | 2 | 0 | 0 | 0 | 2 | 13 | 147 | | 28.15 | 34.80 |
| 21-Jan-1987 | 142 | 0114 | -16.047 | 115.647 | 50 | 66 | 76 | 0 | 0 | 0 | 0 | 15 | 106 | 1076 | | 27.50 | 34.80 |
| 21-Jan-1987 | 143 | 0225 | -16.062 | 115.602 | 50 | 718 | 40 | 3 | 0 | 0 | 0 | 1 | 45 | 58 | | 27.55 | 34.81 |
| 21-Jan-1987 | 144 | 0310 | -16.027 | 115.633 | 50 | 673 | 1 | 0 | 0 | 0 | 0 | 1 | 3 | 152 | | 27.55 | 34.80 |
| 21-Jan-1987 | 145 | 0459 | -16.013 | 115.448 | 50 | 829 | 4 | 4 | 0 | 0 | 0 | 4 | 11 | 124 | | 27.62 | 34.81 |
| 21-Jan-1987 | 146 | 0537 | -16.013 | 115.634 | 50 | 761 | 4 | 0 | 0 | 0 | 0 | 1 | 5 | 217 | | 27.60 | 34.80 |
| 21-Jan-1987 | 147 | 0619 | -15.987 | 115.639 | 50 | 712 | 14 | 0 | 0 | 0 | 0 | 6 | 22 | 174 | | 27.60 | 34.80 |
| 21-Jan-1987 | 148 | 0703 | -15.988 | 115.622 | 50 | 749 | 11 | 3 | 0 | 0 | 0 | 5 | 19 | 128 | | 27.65 | 34.81 |
| 21-Jan-1987 | 149 | 0806 | -16.036 | 115.692 | 50 | 680 | 1 | 0 | 0 | 0 | 0 | 6 | 12 | 141 | | 27.60 | 34.75 |
| 21-Jan-1987 | 151 | 1128 | -15.968 | 115.721 | 50 | 534 | 7 | 2 | 0 | 0 | 0 | 7 | 24 | 401 | | 27.66 | 34.74 |
| 21-Jan-1987 | 152 | 1218 | -15.956 | 115.700 | 50 | 534 | 7 | 0 | 0 | 0 | 0 | 4 | 11 | 577 | | 27.60 | 34.89 |
| 21-Jan-1987 | 153 | 1353 | -15.975 | 115.688 | 50 | 703 | 9 | 0 | 0 | 0 | 0 | 1 | 10 | 441 | | 27.58 | 34.79 |
| 21-Jan-1987 | 154 | 1441 | -15.955 | 115.748 | 50 | 715 | 4 | 0 | 0 | 0 | 0 | 7 | 14 | 505 | | 27.58 | 34.79 |
| 21-Jan-1987 | 155 | 1556 | -15.936 | 115.690 | 50 | 618 | 154 | 2 | 0 | 0 | 0 | 3 | 167 | 534 | | 27.57 | 34.82 |
| 21-Jan-1987 | 156 | 1645 | -15.968 | 115.696 | 50 | 702 | 7 | 0 | 0 | 1 | 0 | 3 | 13 | 239 | | 27.55 | 34.80 |
| 21-Jan-1987 | 157 | 1731 | -15.930 | 115.743 | 50 | 674 | 36 | 12 | 0 | 0 | 0 | 3 | 59 | 435 | | 27.54 | 34.79 |
| 21-Jan-1987 | 158 | 1825 | -15.954 | 115.737 | 50 | 675 | 116 | 1 | 0 | 0 | 0 | 3 | 133 | 486 | | 27.50 | 34.79 |
| 21-Jan-1987 | 159 | 1928 | -15.954 | 115.694 | 50 | 680 | 31 | 0 | 0 | 0 | 0 | 0 | 34 | 497 | | 27.50 | 34.80 |
| 21-Jan-1987 | 160 | 2006 | -15.958 | 115.698 | 50 | 720 | 24 | 3 | 0 | 1 | 0 | 3 | 33 | 511 | | 27.51 | 34.80 |
| 22-Jan-1987 | 162 | 0021 | -15.940 | 115.770 | 50 | 646 | 2 | 2 | 0 | 0 | 0 | 0 | 28 | 228 | | 27.45 | 34.76 |
| 22-Jan-1987 | 163 | 0124 | -15.842 | 115.741 | 50 | 559 | 7 | 2 | 0 | 0 | 0 | 5 | 20 | 179 | | 27.52 | 34.81 |
| 22-Jan-1987 | 164 | 0209 | -15.876 | 115.756 | 50 | 715 | 8 | 0 | 0 | 0 | 0 | 4 | 15 | 97 | | 27.50 | 34.81 |
| 22-Jan-1987 | 165 | 0343 | -15.896 | 115.758 | 50 | 624 | 10 | 2 | 0 | 0 | 0 | 0 | 13 | 313 | | 27.51 | 34.79 |
| 22-Jan-1987 | 166 | 0452 | -15.872 | 115.816 | 50 | 820 | 1 | 0 | 0 | 0 | 0 | 2 | 10 | 99 | | 27.70 | 34.79 |
| 22-Jan-1987 | 167 | 0557 | -15.908 | 115.767 | 50 | 679 | 6 | 10 | 0 | 1 | 0 | 0 | 19 | 284 | | 27.73 | 34.79 |
| 22-Jan-1987 | 168 | 0635 | -15.968 | 115.800 | 50 | 670 | 1 | 0 | 0 | 1 | 0 | 1 | 22 | 146 | | 27.70 | 34.76 |
| 22-Jan-1987 | 169 | 0717 | -15.868 | 115.830 | 50 | 707 | 4 | 3 | 0 | 1 | 0 | 1 | 16 | 355 | | 27.73 | 34.78 |
| 27-Jan-1987 | 177 | 0023 | -15.000 | 116.001 | 30 | 440 | 11 | 2 | 0 | 5 | 0 | 0 | 25 | 193 | | 28.28 | 34.55 |
| 27-Jan-1987 | 178 | 0052 | -14.998 | 115.999 | 40 | 475 | 11 | 4 | 0 | 2 | 0 | 6 | 29 | 288 | | 28.18 | 34.55 |
| 27-Jan-1987 | 179 | 0115 | -15.000 | 116.001 | 40 | 724 | 11 | 8 | 1 | 1 | 0 | 4 | 29 | 423 | | 28.20 | 34.56 |
| 27-Jan-1987 | 180 | 0149 | -15.003 | 115.999 | 40 | 499 | 10 | 12 | 0 | 4 | 0 | 0 | 32 | 379 | | 28.20 | 34.56 |
| 27-Jan-1987 | 181 | 0534 | -15.010 | 116.039 | 40 | 510 | 2 | 0 | 0 | 2 | 0 | 2 | 10 | 192 | | 28.60 | 34.56 |
| 27-Jan-1987 | 182 | 0607 | -14.992 | 116.053 | 40 | 522 | 0 | 0 | 0 | 2 | 0 | 0 | 27 | 224 | | 28.80 | 34.56 |
| 27-Jan-1987 | 183 | 0651 | -14.970 | 116.068 | 40 | 561 | 0 | 9 | 0 | 5 | 0 | 0 | 27 | 305 | | 28.70 | 34.56 |
| 27-Jan-1987 | 184 | 0726 | -14.961 | 116.093 | 40 | 538 | 4 | 4 | 0 | 4 | 0 | 0 | 20 | 284 | | 28.80 | 34.56 |
| 27-Jan-1987 | 185 | 0802 | -14.946 | 116.108 | 40 | 565 | 0 | 0 | 0 | 2 | 0 | 0 | 7 | 485 | | 28.87 | 34.56 |
| 27-Jan-1987 | 186 | 0835 | -14.930 | 116.122 | 40 | 540 | 4 | 4 | 0 | 2 | 0 | 0 | 9 | 270 | | 28.80 | 34.54 |
| 28-Jan-1987 | 193 | 0820 | -16.502 | 115.849 | 40 | 574 | 3279 | 0 | 0 | 26 | 0 | 0 | 5463 | 449 | | 27.95 | 34.84 |
| 29-Jan-1987 | 247 | 0043 | -16.542 | 115.881 | 40 | 311 | 77 | 0 | 0 | 0 | 0 | 0 | 80 | 476 | | 27.50 | 34.82 |
| 29-Jan-1987 | 248 | 0107 | -16.525 | 115.909 | 40 | 336 | 63 | 0 | 0 | 0 | 0 | 0 | 83 | 289 | | 27.50 | 34.82 |
| 29-Jan-1987 | 249 | 0133 | -16.522 | 115.888 | 40 | 520 | 58 | 0 | 0 | 2 | 0 | 0 | 67 | 325 | | 27.50 | 34.82 |
| 29-Jan-1987 | 250 | 0236 | -16.521 | 115.966 | 40 | 711 | 14 | 1 | 0 | 1 | 0 | 0 | 21 | 197 | | 27.52 | 34.84 |
| 29-Jan-1987 | 251 | 0336 | -16.480 | 115.856 | 40 | 710 | 61 | 0 | 0 | 11 | 0 | 0 | 72 | 283 | | 27.55 | 34.84 |
| 29-Jan-1987 | 252 | 0437 | -16.453 | 115.850 | 40 | 583 | 705 | 2 | 0 | 0 | 0 | 2 | 712 | 149 | | 27.60 | 34.84 |
| 29-Jan-1987 | 253 | 0523 | -16.430 | 115.835 | 40 | 336 | 714 | 3 | 0 | 0 | 0 | 3 | 723 | 173 | | 27.70 | 34.87 |
| 29-Jan-1987 | 254 | 0602 | -16.465 | 115.894 | 40 | 327 | 49 | 0 | 0 | 0 | 0 | 0 | 58 | 135 | | 27.80 | 34.88 |
| 29-Jan-1987 | 256 | 1204 | -16.465 | 115.917 | 40 | 240 | 63 | 0 | 4 | 0 | 0 | 0 | 67 | 346 | | 27.65 | 34.82 |
| 29-Jan-1987 | 257 | 1237 | -16.452 | 115.935 | 40 | 331 | 48 | 3 | 0 | 0 | 0 | 3 | 54 | 647 | | 27.65 | 34.85 |
| 29-Jan-1987 | 258 | 1312 | -16.445 | 115.977 | 40 | 661 | 50 | 2 | 0 | 2 | 0 | 2 | 54 | 312 | | 27.65 | 34.87 |
| 29-Jan-1987 | 259 | 1404 | -16.423 | 115.946 | 40 | 490 | 114 | 6 | 0 | 0 | 0 | 8 | 137 | 431 | | 27.61 | 34.86 |
| 29-Jan-1987 | 260 | 1449 | -16.423 | 116.014 | 40 | 256 | 31 | 0 | 0 | 4 | 0 | 4 | 43 | 355 | | 27.62 | 34.88 |
| 29-Jan-1987 | 261 | 1527 | -16.384 | 115.983 | 40 | 501 | 8 | 10 | 2 | 4 | 0 | 2 | 30 | 499 | | 27.30 | 34.70 |
| 29-Jan-1987 | 262 | 1654 | -16.504 | 115.947 | 40 | 347 | 20 | 3 | 0 | 0 | 0 | 9 | 40 | 326 | | 27.60 | 34.86 |
| 29-Jan-1987 | 263 | 1741 | -16.488 | 116.038 | 40 | 709 | 14 | 0 | 0 | 0 | 0 | 3 | 113 | 306 | | 27.55 | 34.83 |
| 29-Jan-1987 | 264 | 1825 | -16.438 | 116.036 | 40 | 310 | 10 | 6 | 0 | 0 | 0 | 0 | 16 | 426 | | 27.60 | 34.86 |
| 29-Jan-1987 | 265 | 1924 | -16.380 | 115.972 | 40 | 663 | 3 | 2 | 3 | 0 | 0 | 2 | 9 | 570 | | 27.40 | 34.64 |
| 30-Jan-1987 | 267 | 0229 | -16.586 | 115.857 | 40 | 356 | 2517 | 11 | 0 | 0 | 0 | 0 | 2528 | 584 | | 27.50 | 34.82 |
| 30-Jan-1987 | 268 | 0256 | -16.582 | 115.884 | 40 | 577 | 80 | 0 | 2 | 0 | 0 | 0 | 90 | 243 | | 27.56 | 34.83 |
| 30-Jan-1987 | 269 | 0332 | -16.576 | 115.907 | 40 | 645 | 5 | 3 | 0 | 0 | 0 | 0 | 8 | 274 | | 27.57 | 34.84 |
| 30-Jan-1987 | 270 | 0437 | -16.603 | 115.903 | 40 | 307 | 33 | 0 | 0 | 3 | 0 | 0 | 36 | 137 | | 27.60 | 34.83 |
| 30-Jan-1987 | 271 | 0523 | -16.544 | 115.898 | 40 | 695 | 26 | 4 | 1 | 3 | 0 | 0 | 35 | 171 | | 27.60 | 34.85 |
| 30-Jan-1987 | 272 | 0617 | -16.540 | 115.844 | 40 | 310 | 987 | 0 | 3 | 3 | 0 | 0 | 994 | 213 | | 27.60 | 34.84 |
| 30-Jan-1987 | 273 | 0654 | -16.526 | 115.932 | 40 | 631 | 25 | 11 | 0 | 5 | 0 | 2 | 60 | 494 | | 27.60 | 34.84 |
| 30-Jan-1987 | 274 | 0801 | -16.458 | 115.841 | 40 | 300 | 573 | 17 | 0 | 7 | 0 | 0 | 603 | 367 | | 27.69 | 34.84 |
| 30-Jan-1987 | 276 | 1235 | -16.617 | 115.819 | 40 | 337 | 463 | 0 | 0 | 6 | 0 | 0 | 593 | 0 | | 27.50 | 34.82 |
| 30-Jan-1987 | 277 | 1307 | -16.629 | 115.886 | 40 | 703 | 549 | 0 | 0 | 0 | 0 | 3 | 569 | 579 | | 27.50 | 34.83 |
| 30-Jan-1987 | 278 | 1346 | -16.588 | 115.902 | 40 | 596 | 23 | 3 | 2 | 0 | 0 | 0 | 29 | 522 | | 27.55 | 34.85 |
| 30-Jan-1987 | 279 | 1429 | -16.547 | 115.930 | 40 | 419 | 2 | 10 | 0 | 0 | 0 | 2 | 14 | 200 | | 27.55 | 34.87 |
| 30-Jan-1987 | 280 | 1520 | -16.549 | 115.877 | 40 | 652 | 365 | 0 | 0 | 0 | 0 | 2 | 393 | 0 | | | 34.83 |
| 30-Jan-1987 | 281 | 1612 | -16.484 | 115.881 | 40 | 329 | 21 | 0 | 0 | 0 | 0 | 3 | 30 | 255 | | 27.45 | 34.83 |
| 30-Jan-1987 | 282 | 1641 | -16.473 | 115.899 | 40 | 611 | 33 | 8 | 0 | 0 | 0 | 0 | 44 | 390 | | 27.45 | 34.82 |
| 30-Jan-1987 | 283 | 1832 | -16.554 | 115.767 | 40 | 310 | 13 | 0 | 0 | 0 | 0 | 3 | 16 | 784 | | 27.45 | 34.86 |
| 30-Jan-1987 | 284 | 1907 | -16.571 | 115.823 | 40 | 352 | 915 | 0 | 0 | 0 | 0 | 0 | 926 | 0 | | 27.50 | 34.86 |
| 30-Jan-1987 | 285 | 2002 | -16.525 | 115.962 | 40 | 569 | 11 | 0 | 0 | 0 | 0 | 2 | 19 | 455 | | 27.50 | 34.85 |
| 31-Jan-1987 | 287 | 0036 | -16.502 | 115.893 | 40 | 306 | 0 | 0 | 0 | 0 | 0 | 0 | 3 | 92 | | 27.42 | 34.84 |
| 31-Jan-1987 | 288 | 0100 | -16.516 | 115.894 | 40 | 585 | 22 | 5 | 3 | 0 | 0 | 2 | 48 | 274 | | 27.45 | 34.85 |
| 31-Jan-1987 | 289 | 0205 | -16.562 | 115.858 | 40 | 624 | 606 | 2 | 0 | 0 | 0 | 0 | 615 | 231 | | 27.46 | 34.83 |
| 31-Jan-1987 | 290 | 0305 | -16.584 | 115.830 | 40 | 330 | 1109 | 3 | 0 | 0 | 0 | 0 | 1127 | 261 | | 27.45 | 34.82 |
| 31-Jan-1987 | 291 | 0329 | -16.599 | 115.839 | 40 | 589 | 1601 | 7 | 0 | 0 | 0 | 0 | 1615 | 263 | | 27.48 | 34.82 |
| 31-Jan-1987 | 292 | 0446 | -16.522 | 115.762 | 40 | 324 | 9 | 0 | 0 | 0 | 0 | 3 | 12 | 123 | | 27.60 | 34.89 |
| 31-Jan-1987 | 293 | 0509 | -16.515 | 115.773 | 40 | 328 | 9 | 0 | 0 | 0 | 0 | 0 | 12 | 238 | | 27.60 | 34.88 |
| 31-Jan-1987 | 294 | 0545 | -16.441 | 115.793 | 40 | 333 | 547 | 0 | 0 | 6 | 0 | 6 | 565 | 273 | | 27.60 | 34.86 |
| 31-Jan-1987 | 295 | 0622 | -16.438 | 115.850 | 40 | 640 | 28 | 3 | 2 | 0 | 0 | 2 | 34 | 0 | | 27.46 | 34.79 |
| 31-Jan-1987 | 296 | 0701 | -16.437 | 115.860 | 40 | 718 | 14 | 1 | 4 | 0 | 0 | 0 | 19 | 304 | | 27.45 | 34.79 |

**Table S1** (continued)

| Date | Stn/Tow # | Time GMT | Latitude | Longitude | Depth (m) | Vol ($m^3$) | SBT | ALB | YFT | ALB/YFT | BET | SKJ | Tot Tuna | Other | MLD (m) | Temp (°C) | Sal (psu) |
|---|---|---|---|---|---|---|---|---|---|---|---|---|---|---|---|---|---|
| 31-Jan-1987 | 298 | 1207 | -16.538 | 115.850 | 40 | 323 | 192 | 9 | 3 | 0 | 0 | 0 | 211 | 3 | | 27.55 | 34.85 |
| 31-Jan-1987 | 299 | 1239 | -16.563 | 115.830 | 40 | 320 | 506 | 6 | 6 | 0 | 0 | 6 | 541 | 244 | | 27.55 | 34.84 |
| 31-Jan-1987 | 300 | 1310 | -16.515 | 115.812 | 40 | 666 | 745 | 3 | 8 | 0 | 0 | 0 | 755 | 411 | | 27.50 | 34.83 |
| 31-Jan-1987 | 301 | 1426 | -16.587 | 115.784 | 40 | 651 | 17 | 6 | 0 | 0 | 0 | 3 | 28 | 611 | | 27.50 | 34.87 |
| 31-Jan-1987 | 302 | 1506 | -16.584 | 115.766 | 40 | 290 | 3 | 0 | 0 | 7 | 0 | 3 | 21 | 221 | | 27.50 | 34.88 |
| 31-Jan-1987 | 303 | 1005 | -16.612 | 115.856 | 40 | 699 | 375 | 0 | 0 | 0 | 0 | 0 | 383 | 289 | | 27.50 | 34.89 |
| 31-Jan-1987 | 304 | 1643 | -16.608 | 115.851 | 40 | 307 | 153 | 0 | 0 | 0 | 0 | 0 | 156 | 195 | | 27.50 | 34.84 |
| 31-Jan-1987 | 305 | 1709 | -16.594 | 115.886 | 40 | 330 | 0 | 6 | 3 | 0 | 0 | 9 | 18 | 248 | | 27.50 | 34.85 |
| 31-Jan-1987 | 306 | 1802 | -16.631 | 115.887 | 40 | 612 | 82 | 2 | 0 | 0 | 0 | 2 | 87 | 337 | | 27.50 | 34.85 |
| 31-Jan-1987 | 307 | 1857 | -16.625 | 115.917 | 40 | 633 | 11 | 6 | 2 | 0 | 0 | 2 | 24 | 299 | | 27.50 | 34.85 |
| 1-Feb-1987 | 308 | 0055 | -16.525 | 115.852 | 40 | 538 | 9 | 0 | 0 | 2 | 0 | 0 | 17 | 223 | | 27.30 | 34.78 |
| 1-Feb-1987 | 310 | 0258 | -16.500 | 115.812 | 40 | 335 | 290 | 6 | 0 | 0 | 0 | 3 | 299 | 137 | | 27.45 | 34.84 |
| 1-Feb-1987 | 311 | 0316 | -16.500 | 115.845 | 40 | 341 | 264 | 3 | 0 | 0 | 0 | 0 | 279 | 267 | | 27.45 | 34.83 |
| 1-Feb-1987 | 312 | 0447 | -16.400 | 115.864 | 40 | 603 | 2 | 3 | 0 | 3 | 0 | 0 | 12 | 216 | | 27.18 | 34.68 |
| 1-Feb-1987 | 313 | 0535 | -16.453 | 115.908 | 40 | 359 | 0 | 8 | 0 | 0 | 0 | 6 | 17 | 540 | | 27.20 | 34.72 |
| 1-Feb-1987 | 314 | 0556 | -16.457 | 115.907 | 40 | 674 | 0 | 3 | 0 | 0 | 0 | 7 | 12 | 493 | | 27.20 | 34.72 |
| 1-Feb-1987 | 315 | 0626 | -16.434 | 115.928 | 40 | 631 | 5 | 3 | 0 | 3 | 0 | 0 | 11 | 409 | | 27.23 | 34.74 |
| 1-Feb-1987 | 316 | 0723 | -16.500 | 115.929 | 40 | 301 | 0 | 0 | 0 | 0 | 0 | 0 | 0 | 146 | | 27.40 | 34.82 |
| 1-Feb-1987 | 317 | 0805 | -16.565 | 115.886 | 40 | 295 | 3 | 0 | 0 | 0 | 0 | 0 | 3 | 397 | | 27.49 | 34.84 |
| 1-Feb-1987 | 318 | 0824 | -16.557 | 115.904 | 40 | 646 | 0 | 6 | 0 | 0 | 0 | 2 | 12 | 234 | | 27.49 | 34.84 |
| 1-Feb-1987 | 320 | 1228 | -16.517 | 115.883 | 40 | 283 | 14 | 7 | 0 | 7 | 0 | 0 | 32 | 346 | | 27.35 | 34.82 |
| 1-Feb-1987 | 321 | 1331 | -16.466 | 115.854 | 40 | 643 | 19 | 11 | 2 | 6 | 0 | 5 | 42 | 435 | | 27.25 | 34.76 |
| 1-Feb-1987 | 322 | 1406 | -16.454 | 115.850 | 40 | 303 | 0 | 3 | 0 | 0 | 0 | 0 | 10 | 465 | | 27.20 | 34.74 |
| 1-Feb-1987 | 323 | 1441 | -16.458 | 115.905 | 40 | 352 | 0 | 0 | 0 | 6 | 0 | 3 | 11 | 330 | | 27.29 | 34.78 |
| 1-Feb-1987 | 324 | 1527 | -16.479 | 115.911 | 40 | 323 | 3 | 6 | 0 | 0 | 0 | 0 | 9 | 226 | | 27.30 | 34.80 |
| 1-Feb-1987 | 325 | 1641 | -16.484 | 115.835 | 40 | 655 | 50 | 5 | 0 | 0 | 0 | 8 | 64 | 412 | | 27.25 | 34.78 |
| 1-Feb-1987 | 326 | 1742 | -16.534 | 115.850 | 40 | 631 | 166 | 8 | 3 | 0 | 0 | 0 | 190 | 610 | | 27.39 | 34.82 |
| 1-Feb-1987 | 327 | 1846 | -16.464 | 115.804 | 40 | 601 | 32 | 0 | 0 | 2 | 0 | 5 | 43 | 404 | | 27.15 | 34.74 |
| 1-Feb-1987 | 328 | 1933 | -16.384 | 115.825 | 40 | 282 | 4 | 0 | 0 | 0 | 0 | 0 | 7 | 337 | | 27.05 | 34.68 |
| 1-Feb-1987 | 329 | 2000 | -16.395 | 115.867 | 40 | 630 | 10 | 6 | 0 | 0 | 0 | 2 | 19 | 456 | | 27.00 | 34.67 |
| 2-Feb-1987 | 331 | 0119 | -16.477 | 115.867 | 40 | 710 | 1 | 6 | 0 | 3 | 0 | 0 | 10 | 187 | | 27.20 | 34.78 |
| 2-Feb-1987 | 332 | 0215 | -16.472 | 115.921 | 40 | 647 | 5 | 0 | 0 | 0 | 0 | 2 | 8 | 139 | | 27.25 | 34.80 |
| 2-Feb-1987 | 333 | 0311 | -16.454 | 115.917 | 40 | 677 | 1 | 0 | 0 | 0 | 0 | 0 | 1 | 133 | | 27.20 | 34.78 |
| 2-Feb-1987 | 334 | 0343 | -16.440 | 115.930 | 40 | 345 | 0 | 6 | 0 | 0 | 0 | 0 | 6 | 174 | | 27.19 | 34.75 |
| 2-Feb-1987 | 335 | 0542 | -16.466 | 115.812 | 40 | 358 | 8 | 6 | 0 | 0 | 0 | 0 | 14 | 346 | | 27.40 | 34.77 |
| 2-Feb-1987 | 336 | 0706 | -16.493 | 115.914 | 40 | 336 | 0 | 0 | 0 | 0 | 0 | 3 | 3 | 193 | | 27.40 | 34.79 |
| 2-Feb-1987 | 337 | 0805 | -16.469 | 115.840 | 40 | 351 | 6 | 0 | 0 | 0 | 0 | 6 | 11 | 299 | | 27.20 | 34.75 |
| 2-Feb-1987 | 338 | 0833 | -16.435 | 115.850 | 40 | 664 | 3 | 5 | 2 | 0 | 0 | 0 | 11 | 261 | | 27.20 | 34.75 |
| 2-Feb-1987 | 340 | 1215 | -16.566 | 115.858 | 40 | 390 | 208 | 0 | 0 | 0 | 0 | 0 | 215 | 472 | | 27.30 | 34.75 |
| 2-Feb-1987 | 341 | 1301 | -16.555 | 115.835 | 40 | 639 | 221 | 3 | 3 | 0 | 2 | 0 | 233 | 178 | | 27.25 | 34.83 |
| 2-Feb-1987 | 342 | 1351 | -16.559 | 115.675 | 40 | 352 | 253 | 3 | 6 | 0 | 0 | 0 | 276 | 139 | | 27.25 | 34.83 |
| 2-Feb-1987 | 343 | 1434 | -16.600 | 115.817 | 40 | 661 | 241 | 5 | 2 | 0 | 0 | 0 | 256 | 151 | | 27.25 | 34.83 |
| 2-Feb-1987 | 344 | 1531 | -16.486 | 115.812 | 40 | 658 | 29 | 0 | 0 | 0 | 0 | 2 | 47 | 150 | | 27.20 | 34.81 |
| 31-Jan-2022 | 1 | 1147 | -13.238 | 119.466 | 25 | 462 | 26 | 0 | 0 | | 2 | 0 | 28 | | 40 | 29.09 | 34.24 |
| 2-Feb-2022 | 2 | 0510 | -13.256 | 119.115 | 25 | 558 | 0 | 0 | 2 | | 0 | 2 | 4 | 104 | | | |
| 2-Feb-2022 | 3 | 2316 | -13.503 | 114.366 | 25 | 520 | 6 | 0 | 2 | | 0 | 0 | 8 | 158 | 61 | 29.18 | 34.26 |
| 2-Feb-2022 | 6 | 0811 | -13.504 | 113.967 | 25 | 432 | 5 | | | | | | | | 8 | 29.33 | 34.20 |
| 2-Feb-2022 | 8 | 1044 | -14.070 | 114.428 | 30 | 709 | 3 | | | | | | | | | | |
| 2-Feb-2022 | 9 | 1326 | -14.334 | 114.664 | 30 | 640 | 9 | | | | | | | | 29 | 29.18 | 34.30 |
| 3-Feb-2022 | 10 | 0449 | -15.391 | 114.685 | 30 | 497 | 105 | 4 | 10 | | 0 | 0 | 123 | 237 | 36 | 28.60 | 34.51 |
| 3-Feb-2022 | 11 | 0450 | -15.486 | 114.715 | 30 | 565 | 14 | 2 | 3 | | 0 | 0 | 35 | 117 | | | |
| 3-Feb-2022 | 12 | 1005 | -15.369 | 114.652 | 30 | 480 | 48 | 4 | 13 | | 4 | 0 | 69 | 148 | 32 | 28.67 | 34.51 |
| 3-Feb-2022 | 14 | 1626 | -15.379 | 114.599 | 30 | 632 | 9 | 3 | 2 | | 0 | 2 | 16 | 87 | 38 | 28.61 | 34.52 |
| 3-Feb-2022 | 15 | 2142 | -15.342 | 114.518 | 30 | 782 | 6 | 3 | 0 | | 0 | 3 | 23 | 233 | 44 | 28.59 | 34.52 |
| 4-Feb-2022 | 16 | 0334 | -15.334 | 114.500 | 30 | 498 | 14 | 0 | 0 | | 0 | 0 | 14 | 0 | 42 | 28.59 | 34.52 |
| 4-Feb-2022 | 17 | 0336 | -15.342 | 114.477 | 30 | 732 | 34 | 3 | 5 | | 0 | 0 | 48 | 292 | 12 | 28.61 | 34.53 |
| 4-Feb-2022 | 18 | 0829 | -15.350 | 114.520 | 30 | 726 | 25 | 1 | 6 | | 0 | 0 | 33 | 121 | 44 | 28.61 | 34.53 |
| 4-Feb-2022 | 19 | 1626 | -15.360 | 114.420 | 30 | 723 | 18 | 4 | 7 | | 3 | 1 | 33 | 321 | 40 | 28.60 | 34.53 |
| 4-Feb-2022 | 20 | 1627 | -15.370 | 114.400 | 30 | 685 | 7 | 6 | 0 | | 0 | 0 | 13 | 99 | 42 | 28.58 | 34.53 |
| 4-Feb-2022 | 21 | 2040 | -15.375 | 114.368 | 30 | 863 | 5 | 1 | 0 | | 0 | 2 | 10 | 95 | 48 | 28.54 | 34.54 |
| 5-Feb-2022 | 22 | 0117 | -15.367 | 114.364 | 30 | 362 | 11 | 0 | 3 | | 0 | 0 | 17 | 127 | 49 | 28.54 | 34.54 |
| 5-Feb-2022 | 23 | 0335 | -15.358 | 114.341 | 30 | 399 | 6 | 10 | 4 | | 0 | 0 | 22 | 84 | 23 | 28.56 | 34.55 |
| 5-Feb-2022 | 24 | 1250 | -15.370 | 114.303 | 30 | 634 | 10 | 5 | 0 | | 0 | 0 | 31 | 141 | 9 | 28.59 | 34.55 |
| 5-Feb-2022 | 25 | 1253 | -15.387 | 114.259 | 30 | 561 | 9 | 1 | 0 | | 2 | 0 | 13 | 307 | 21 | 28.58 | 34.55 |
| 5-Feb-2022 | 26 | 1300 | -15.375 | 114.274 | 30 | 716 | 13 | 3 | 6 | | 0 | 1 | 31 | 338 | 36 | 28.58 | 34.55 |
| 5-Feb-2022 | 28 | 1551 | -15.370 | 114.260 | 30 | 718 | 191 | 22 | 0 | | 0 | 0 | 227 | 316 | 54 | 28.52 | 34.55 |
| 5-Feb-2022 | 30 | 2037 | -15.401 | 114.233 | 30 | 391 | 69 | 13 | 3 | | 0 | 0 | 84 | 235 | 49 | 28.53 | 34.56 |
| 6-Feb-2022 | 31 | 0059 | -15.411 | 114.218 | 42 | 787 | 9 | 1 | 1 | | 0 | 0 | 11 | 103 | 49 | 28.52 | 34.56 |
| 6-Feb-2022 | 32 | 0335 | -15.419 | 114.212 | 33 | 719 | 8 | 1 | 1 | | 0 | 0 | 12 | 47 | 7 | 28.54 | 34.50 |
| 6-Feb-2022 | 33 | 0725 | -15.440 | 114.190 | 25 | 649 | 6 | 2 | 2 | | 0 | 0 | 9 | 174 | 7 | 28.60 | 34.51 |
| 6-Feb-2022 | 34 | 1354 | -15.460 | 114.190 | 36 | 536 | 0 | 2 | 0 | | 0 | 0 | 2 | 222 | 6 | 28.57 | 34.47 |
| 6-Feb-2022 | 36 | 1523 | -15.470 | 114.170 | 42 | 666 | 11 | 2 | 0 | | 0 | 2 | 14 | 281 | 23 | 28.58 | 34.54 |
| 6-Feb-2022 | 37 | 2010 | -15.470 | 114.190 | 42 | 737 | 26 | 20 | 5 | | 0 | 0 | 62 | 187 | 30 | 28.57 | 34.54 |
| 7-Feb-2022 | 38 | 0022 | -15.478 | 114.173 | 42 | 675 | 24 | 4 | 4 | | 0 | 0 | 33 | 256 | 49 | 28.54 | 34.54 |
| 7-Feb-2022 | 39 | 0344 | -15.488 | 114.164 | 42 | 532 | 61 | 21 | 14 | | 0 | 0 | 126 | 149 | 6 | 28.78 | 34.54 |
| 7-Feb-2022 | 40 | 0853 | -15.520 | 114.140 | 42 | 632 | 3 | 3 | 2 | | 0 | 0 | 8 | 158 | | | |
| 7-Feb-2022 | 43 | 1932 | -15.591 | 114.134 | 41 | 743 | 11 | 3 | 0 | | 0 | 0 | 23 | 230 | 26 | 28.64 | 34.55 |
| 8-Feb-2022 | 45 | 0509 | -15.800 | 114.500 | 22 | 701 | 24 | | | | | | | | 8 | 28.74 | 34.53 |
| 8-Feb-2022 | 46 | 1408 | -15.970 | 114.770 | 16 | 782 | 14 | | | | | | | | 7 | 29.06 | 34.52 |
| 8-Feb-2022 | 47 | 1410 | -16.230 | 115.030 | 16 | 812 | 62 | 0 | 0 | | 0 | 1 | 75 | | 18 | 28.41 | 34.77 |
| 8-Feb-2022 | 48 | 1803 | -16.464 | 115.287 | 21 | 750 | 4 | 0 | 0 | | 0 | 8 | 15 | 849 | 11 | 28.37 | 34.85 |
| 8-Feb-2022 | 49 | 2220 | -16.711 | 115.558 | 26 | 646 | 77 | | | | | | | | | | |
| 9-Feb-2022 | 50 | 0138 | -16.948 | 115.801 | 19 | 523 | 4 | | | | | | | | 22 | 28.41 | 34.72 |
| 9-Feb-2022 | 51 | 0531 | -17.170 | 116.020 | 21 | 710 | 14 | 3 | 11 | | 0 | 0 | 35 | 90 | 8 | 29.06 | 34.94 |
| 9-Feb-2022 | 52 | 1739 | -17.115 | 115.983 | 25 | 623 | 3 | | | | | | | | 7 | 28.29 | 34.85 |
| 9-Feb-2022 | 55 | 1755 | -16.733 | 115.691 | 25 | 715 | 22 | | | | | | | | | | |
| 9-Feb-2022 | 56 | 2112 | -16.749 | 115.711 | 25 | 698 | 13 | 0 | 0 | | 0 | 1 | 14 | 350 | 11 | 28.48 | 34.83 |
| 10-Feb-2022 | 57 | 0113 | -16.739 | 115.755 | 30 | 673 | 5 | 0 | 0 | | 0 | 1 | 8 | 554 | 14 | 28.28 | 34.84 |
| 10-Feb-2022 | 58 | 0346 | -16.746 | 115.761 | 28 | 586 | 1 | 0 | 0 | | 0 | 2 | 3 | 321 | 8 | 28.73 | 34.77 |
| 10-Feb-2022 | 59 | 0733 | -16.730 | 115.760 | 28 | 750 | 5 | 0 | 0 | | 0 | 1 | 7 | 360 | 6 | 28.47 | 34.83 |
| 10-Feb-2022 | 62 | 1556 | -16.770 | 115.790 | 37 | 631 | 17 | 0 | 0 | | 0 | 2 | 19 | 304 | | | |
| 10-Feb-2022 | 63 | 2053 | -16.817 | 115.832 | 29 | 585 | 7 | | | | | | | | 7 | 28.83 | 34.84 |
| 11-Feb-2022 | 64 | 0056 | -16.834 | 115.850 | 28 | 565 | 7 | 0 | 0 | | 0 | 0 | 7 | 380 | 16 | 28.74 | 34.85 |
| 11-Feb-2022 | 65 | 0418 | -16.834 | 115.881 | 22 | 561 | 10 | 0 | 0 | | 0 | 1 | 12 | 154 | 6 | 28.98 | 34.85 |

**Table S1** (continued)

| Date | Stn/Tow # | Time GMT | Latitude | Longitude | Depth (m) | Vol ($m^3$) | SBT | ALB | YFT | ALB/YFT | BET | SKJ | Tot Tuna | Other | MLD (m) | Temp (°C) | Sal (psu) |
|---|---|---|---|---|---|---|---|---|---|---|---|---|---|---|---|---|---|
| 11-Feb-2022 | 66 | 0722 | -16.820 | 115.900 | 21 | 591 | 10 | 0 | 1 | | 0 | 0 | 11 | 137 | 6 | 29.03 | 34.86 |
| 11-Feb-2022 | 67 | 1108 | -16.850 | 115.920 | 22 | 601 | 8 | 0 | 0 | | 0 | 0 | 8 | 617 | 6 | 28.72 | 34.83 |
| 11-Feb-2022 | 69 | 1556 | -16.860 | 115.930 | 19 | 613 | 15 | 0 | 2 | | 0 | 11 | 29 | 241 | 6 | 28.94 | 34.84 |
| 11-Feb-2022 | 70 | 2051 | -16.859 | 115.934 | 27 | 679 | 7 | | | | | | | | 18 | 28.93 | 34.84 |
| 12-Feb-2022 | 71 | 0041 | -16.922 | 115.949 | 29 | 505 | 7 | 1 | 1 | | 0 | 0 | 9 | 507 | 12 | 28.84 | 34.83 |
| 12-Feb-2022 | 72 | 0339 | -16.941 | 115.962 | 25 | 558 | 14 | 0 | 1 | | 0 | 0 | 16 | 92 | 6 | 29.15 | 34.79 |
| 12-Feb-2022 | 73 | 0731 | -16.953 | 115.995 | 31 | 450 | 10 | 0 | 1 | | 0 | 0 | 11 | 177 | 6 | 29.20 | 34.75 |
| 12-Feb-2022 | 74 | 1226 | -16.996 | 116.039 | 20 | 557 | 7 | 0 | 0 | | 0 | 4 | 11 | 406 | 6 | 29.02 | 34.81 |
| 12-Feb-2022 | 77 | 1610 | -17.008 | 116.054 | 17 | 616 | 11 | 0 | 0 | | 0 | 5 | 16 | 114 | 12 | 29.06 | 34.84 |
| 12-Feb-2022 | 78 | 2123 | -16.998 | 116.072 | 20 | 675 | 7 | | | | | | | | 22 | 28.84 | 34.84 |
| 13-Feb-2022 | 79 | 0110 | -17.010 | 116.073 | 34 | 602 | 8 | 0 | 0 | | 0 | 0 | 8 | 284 | 11 | 28.84 | 34.81 |
| 13-Feb-2022 | 80 | 0427 | -17.064 | 116.084 | 27 | 563 | 3 | 0 | 0 | | 0 | 0 | 3 | 336 | 8 | 28.88 | 34.83 |
| 13-Feb-2022 | 81 | 0735 | -17.060 | 116.101 | 21 | 525 | 5 | 0 | 1 | | 0 | 0 | 10 | 176 | 6 | 29.14 | 34.81 |
| 13-Feb-2022 | 82 | 1222 | -17.070 | 116.113 | 14 | 587 | 5 | 0 | 0 | | 0 | 0 | 5 | 261 | 9 | 29.16 | 34.74 |
| 13-Feb-2022 | 89 | 1532 | -17.099 | 116.124 | 19 | 630 | 2 | 0 | 0 | | 0 | 0 | 3 | 168 | 11 | 28.98 | 34.82 |
| 14-Feb-2022 | 90 | 0721 | -16.090 | 115.590 | 18 | 520 | 17 | | | | | | | | 6 | 29.48 | 34.54 |
| 14-Feb-2022 | 92 | 1147 | -16.010 | 115.590 | 22 | 482 | 21 | | | | | | | | 6 | 29.50 | 34.54 |
| 14-Feb-2022 | 94 | 1323 | -16.100 | 115.520 | 19 | 498 | 14 | | | | | | | | 6 | 29.38 | 34.54 |
| 14-Feb-2022 | 96 | 1610 | -16.160 | 115.660 | 13 | 589 | 2 | | | | | | | | 6 | 29.44 | 34.55 |
| 14-Feb-2022 | 98 | 1926 | -16.062 | 115.773 | 23 | 577 | 17 | | | | | | | | 6 | 29.56 | 34.54 |
| 14-Feb-2022 | 100 | 2246 | -15.861 | 115.683 | 25 | 542 | 2 | | | | | | | | 6 | 29.32 | 34.53 |
| 15-Feb-2022 | 101 | 0256 | -16.020 | 115.708 | 28 | 567 | 4 | | | | | | | | 6 | 29.55 | 34.54 |
| 15-Feb-2022 | 102 | 0258 | -16.020 | 115.800 | 25 | 590 | 17 | | | | | | | | 6 | 29.55 | 34.54 |
| 15-Feb-2022 | 104 | 0617 | -16.000 | 115.900 | 24 | 498 | 22 | | | | | | | | 6 | 29.45 | 34.54 |
| 15-Feb-2022 | 107 | 1141 | -15.980 | 115.830 | 20 | 538 | 52 | 0 | 0 | | 0 | 0 | 52 | 766 | 9 | 29.71 | 34.54 |
| 15-Feb-2022 | 108 | 1636 | -15.980 | 115.830 | 20 | 488 | 47 | 0 | 0 | | 0 | 2 | 49 | 582 | 6 | 29.93 | 34.55 |
| 15-Feb-2022 | 109 | 2023 | -15.943 | 115.830 | 21 | 663 | 14 | 0 | 0 | | 0 | 3 | 18 | 621 | 8 | 29.44 | 34.53 |
| 16-Feb-2022 | 110 | 0048 | -15.938 | 115.827 | 25 | 594 | 19 | 0 | 0 | | 0 | 0 | 19 | 487 | 6 | 29.32 | 34.55 |
| 16-Feb-2022 | 111 | 0329 | -15.938 | 115.830 | 19 | 674 | 25 | 0 | 0 | | 0 | 0 | 36 | 508 | 6 | 29.81 | 34.55 |
| 16-Feb-2022 | 112 | 0657 | -15.920 | 115.830 | 20 | 568 | 57 | 0 | 0 | | 0 | 0 | 57 | 257 | 6 | 29.81 | 34.55 |
| 16-Feb-2022 | 113 | 0855 | -15.910 | 115.830 | 18 | 653 | 15 | 0 | 2 | | 0 | 0 | 20 | 729 | 6 | 29.74 | 34.54 |
| 16-Feb-2022 | 115 | 1417 | -15.900 | 115.830 | 20 | 627 | 35 | 0 | 0 | | 0 | 0 | 37 | 646 | 6 | 29.98 | 34.55 |
| 16-Feb-2022 | 118 | 1959 | -15.838 | 115.838 | 21 | 726 | 12 | 1 | 1 | | 1 | 0 | 22 | 997 | 6 | 29.93 | 34.56 |
| 16-Feb-2022 | 119 | 2324 | -15.857 | 115.848 | 29 | 585 | 27 | 0 | 2 | | 0 | 2 | 34 | 345 | 6 | 29.57 | 34.55 |
| 17-Feb-2022 | 121 | 0329 | -15.842 | 115.846 | 25 | 621 | 31 | 0 | 0 | | 0 | 0 | 31 | -31 | 6 | 29.82 | 34.55 |
| 17-Feb-2022 | 122 | 0717 | -15.824 | 115.831 | 17 | 642 | 23 | 2 | 0 | | 0 | 0 | 25 | 324 | 6 | 29.61 | 34.54 |
| 17-Feb-2022 | 124 | 1244 | -15.805 | 115.821 | 25 | 636 | 57 | 0 | 0 | | 0 | 0 | 57 | 654 | 6 | 30.04 | 34.56 |
| 17-Feb-2022 | 125 | 1541 | -15.781 | 115.817 | 25 | 544 | 9 | 0 | 0 | | 0 | 2 | 11 | 776 | 6 | 29.68 | 34.55 |
| 17-Feb-2022 | 127 | 0194 | -15.763 | 115.800 | 27 | 564 | 28 | 0 | 0 | | 0 | 2 | 30 | 119 | 6 | 30.03 | 34.58 |
| 17-Feb-2022 | 128 | 2351 | -15.745 | 115.797 | 42 | 467 | 90 | 2 | 0 | | 0 | 2 | 96 | 360 | 6 | 30.12 | 34.59 |
| 18-Feb-2022 | 129 | 0326 | -15.735 | 115.799 | 20 | 617 | 71 | 0 | 0 | | 0 | 1 | 74 | 241 | 8 | 30.29 | 34.59 |
| 18-Feb-2022 | 130 | 0718 | -15.704 | 115.775 | 25 | 665 | 60 | 0 | 2 | | 0 | 0 | 62 | 192 | 6 | 30.11 | 34.59 |
| 18-Feb-2022 | 131 | 1215 | -15.690 | 115.774 | 17 | 719 | 35 | 0 | 0 | | 0 | 0 | 35 | 349 | 6 | 30.53 | 34.60 |
| 18-Feb-2022 | 133 | 1438 | -15.646 | 115.785 | 23 | 593 | 103 | 0 | 0 | | 0 | 0 | 105 | 530 | 6 | 30.12 | 34.58 |
| 18-Feb-2022 | 135 | 1853 | -15.620 | 115.774 | 24 | 640 | 84 | 0 | 2 | | 0 | 0 | 89 | 1167 | 7 | 30.19 | 34.59 |
| 19-Feb-2022 | 137 | 0809 | -15.876 | 116.501 | 23 | 618 | 5 | 0 | 0 | | 0 | 0 | 5 | | 8 | 30.19 | 34.57 |
| 19-Feb-2022 | 138 | 1411 | -15.913 | 117.000 | 27 | 591 | 195 | 0 | 0 | | 0 | 2 | 196 | | 13 | 30.43 | 34.59 |
| 19-Feb-2022 | 139 | 1935 | -15.959 | 117.498 | 27 | 602 | 85 | | | | | | | | 6 | 30.28 | 34.53 |
| 19-Feb-2022 | 140 | 2305 | -16.000 | 118.000 | 32 | 488 | 6 | | | | | | | | 10 | 30.20 | 34.56 |
| 20-Feb-2022 | 141 | 0003 | -15.942 | 118.050 | 24 | 415 | 22 | | | | | | | | | | |
| 20-Feb-2022 | 142 | 0302 | -15.883 | 118.105 | 25 | 491 | 153 | | | | | | | | | | |
| 20-Feb-2022 | 145 | 0817 | -15.888 | 118.116 | 17 | 658 | 158 | | | | | | | | 7 | 30.34 | 34.55 |
| 20-Feb-2022 | 146 | 1213 | -15.888 | 118.139 | 38 | 495 | 160 | 0 | 0 | | 0 | 0 | 160 | 584 | 6 | 30.56 | 34.56 |
| 20-Feb-2022 | 147 | 1609 | -15.887 | 118.141 | 28 | 575 | 151 | | | | | | | | 7 | 30.57 | 34.56 |
| 20-Feb-2022 | 148 | 1951 | -15.885 | 118.142 | 18 | 547 | 185 | | | | | | | | 12 | 30.50 | 34.56 |
| 20-Feb-2022 | 149 | 2342 | -15.882 | 118.148 | 28 | 580 | 141 | 0 | 0 | | 0 | 0 | 141 | 178 | 6 | 30.06 | 34.54 |
| 21-Feb-2022 | 150 | 0336 | -15.890 | 118.145 | 30 | 572 | 49 | 0 | 0 | | 0 | 0 | 53 | 54 | 11 | 30.55 | 34.57 |
| 21-Feb-2022 | 151 | 0741 | -15.889 | 118.131 | 37 | 513 | 115 | 0 | 0 | | 0 | 0 | 121 | 162 | 6 | 30.82 | 34.58 |
| 21-Feb-2022 | 152 | 1205 | -15.898 | 118.098 | 23 | 654 | 153 | | | | | | | | 7 | 30.67 | 34.57 |
| 21-Feb-2022 | 154 | 1546 | -15.908 | 118.094 | 18 | 551 | 89 | 0 | 0 | | 0 | 0 | 89 | 332 | 7 | 30.30 | 34.55 |
| 21-Feb-2022 | 155 | 1951 | -15.913 | 118.092 | 17 | 676 | 62 | | | | | | | | 12 | 30.53 | 34.57 |
| 22-Feb-2022 | 156 | 0008 | -15.931 | 118.085 | 23 | 568 | 79 | 0 | 0 | | 4 | 0 | 83 | 153 | 11 | 30.40 | 34.57 |
| 22-Feb-2022 | 157 | 0327 | -15.943 | 118.081 | 24 | 583 | 88 | 0 | 0 | | 0 | 0 | 89 | 57 | 10 | 30.46 | 34.57 |
| 22-Feb-2022 | 158 | 0718 | -15.954 | 118.105 | 34 | 553 | 99 | 0 | 0 | | 0 | 0 | 99 | 94 | 6 | 30.39 | 34.57 |
| 22-Feb-2022 | 159 | 1242 | -15.954 | 118.114 | 24 | 522 | 80 | | | | | | | | 6 | 30.46 | 34.57 |
| 22-Feb-2022 | 161 | 1555 | -15.955 | 118.122 | 25 | 523 | 111 | 0 | 0 | | 0 | 0 | 117 | 465 | 12 | 30.63 | 34.58 |
| 22-Feb-2022 | 162 | 1945 | -15.940 | 118.118 | 24 | 646 | 76 | | | | | | | | 16 | 30.55 | 34.58 |
| 22-Feb-2022 | 163 | 2348 | -15.939 | 118.116 | 20 | 488 | 70 | 0 | 0 | | 0 | 0 | 70 | 100 | 9 | 30.25 | 34.56 |
| 23-Feb-2022 | 164 | 0329 | -15.949 | 118.110 | 18 | 572 | 23 | 0 | 0 | | 0 | 0 | 23 | 74 | 7 | 30.56 | 34.58 |
| 23-Feb-2022 | 165 | 1323 | -15.965 | 118.084 | 19 | 682 | 54 | 0 | 0 | | 0 | 0 | 54 | 339 | 6 | 30.68 | 34.58 |
| 23-Feb-2022 | 166 | 1830 | -15.982 | 118.086 | 19 | 675 | 56 | 0 | 0 | | 0 | 0 | 56 | 293 | 7 | 30.43 | 34.58 |
| 24-Feb-2022 | 168 | 0850 | -15.998 | 119.003 | 18 | 700 | 13 | 0 | 0 | | 0 | 0 | 16 | | 11 | 30.66 | 34.56 |
| 24-Feb-2022 | 169 | 1619 | -14.999 | 119.499 | 22 | 629 | 43 | 0 | 0 | | 0 | 5 | 48 | | 14 | 30.50 | 34.63 |
| 25-Feb-2022 | 170 | 0002 | -15.001 | 118.497 | 26 | 543 | 109 | 0 | 0 | | 0 | 0 | 110 | | 22 | 30.00 | 34.52 |
| 25-Feb-2022 | 171 | 1300 | -15.014 | 117.512 | 21 | 731 | 54 | | | | | | | | 6 | 30.51 | 34.60 |
| 25-Feb-2022 | 172 | 2116 | -15.014 | 116.507 | 18 | 598 | 28 | | | | | | | | 13 | 30.24 | 34.58 |
| 26-Feb-2022 | 173 | 0401 | -15.013 | 115.495 | 25 | 574 | 143 | 0 | 3 | | 3 | 0 | 152 | | 14 | 30.12 | 34.53 |
| 26-Feb-2022 | 176 | 1632 | -15.037 | 114.528 | 22 | 572 | 16 | | | | | | | | | | |
| 27-Feb-2022 | 177 | 0041 | -14.010 | 114.499 | 28 | 562 | 60 | | | | | | | | 15 | 30.18 | 34.41 |
| 27-Feb-2022 | 178 | 0730 | -13.003 | 114.501 | 26 | 579 | 136 | | | | | | | | 17 | 30.05 | 34.23 |
| 27-Feb-2022 | 180 | 2340 | -13.502 | 115.497 | 22 | 529 | 0 | 0 | 0 | | 0 | 0 | 2 | 284 | 29 | 29.99 | 34.41 |
| 28-Feb-2022 | 181 | 0628 | -13.501 | 116.501 | 21 | 585 | 17 | | | | | | | | 9 | 29.98 | 34.43 |
| 28-Feb-2022 | 183 | 1901 | -13.516 | 117.506 | 26 | 647 | 2 | 2 | 0 | | 0 | 9 | 12 | | 26 | 29.97 | 34.38 |
| 1-Mar-2022 | 184 | 0202 | -13.502 | 118.493 | 20 | 632 | 2 | 0 | 0 | | 0 | 0 | 3 | | 28 | 29.66 | 34.44 |
| 1-Mar-2022 | 185 | 0819 | -13.500 | 119.500 | 28 | 602 | 32 | 5 | 3 | | 0 | 0 | 42 | | 22 | 29.64 | 34.31 |
| 1-Mar-2022 | 187 | 2101 | -13.500 | 121.500 | 30 | 1031 | 0 | 0 | 0 | | 0 | 1 | 3 | | 44 | 29.44 | 34.24 |
| 2-Mar-2022 | 188 | 0807 | -13.500 | 121.502 | 33 | 635 | 2 | 0 | 0 | | 0 | 0 | 3 | | 28 | 29.40 | 34.34 |

**Table S2.** Ingested prey of SBT larvae (number/larva) from stomach content analyses from 2022 Argo Basin samples. ID is specimen number from cruise RR2201. Cil = ciliate; Naup = copepod nauplius; Cal = calanoid copepodite; Cory = Corycaeidae (cyclopoid) copepodite; Clad = cladocera; App = appendicularian; Fish = larval fish; Tolal = total prey per stomach.

| | | Prey types in stomach contents | | | | | | | | |
|---|---|---|---|---|---|---|---|---|---|---|
| **ID number** | **Lifestage** | **Cil** | **Naup** | **Cal** | **Cory** | **Clad** | **App** | **Fish** | **Other** | **Total** |
| R2201ET0055 | preflexion | | 1 | | | | | | | 1 |
| R2201ET0057 | preflexion | 3 | 1 | | | | 1 | | | 5 |
| R2201ET0058 | preflexion | | | 1 | | 2 | 2 | | | 5 |
| R2201ET0065 | preflexion | 1 | 1 | | | | | | | 2 |
| R2201ET0066 | preflexion | | | | | | 2 | | 1 | 3 |
| R2201ET0067 | preflexion | | 2 | | | | 1 | | | 3 |
| R2201ET0071 | preflexion | | 1 | | | | | | | 1 |
| R2201ET0080 | preflexion | | | 1 | 1 | | 1 | | | 3 |
| R2201ET0082 | preflexion | | 1 | | | | 3 | | | 4 |
| R2201ET0086 | preflexion | | 1 | | | | 2 | | | 3 |
| R2201ET0087 | preflexion | | 3 | | | | 1 | | | 4 |
| R2201ET0092 | preflexion | | 6 | | | | | | | 6 |
| R2201ET0109 | preflexion | | 3 | | | | 1 | | | 4 |
| R2201ET0201 | preflexion | | 1 | | | | 1 | | | 2 |
| R2201ET0265 | preflexion | | 1 | | | | 1 | | | 2 |
| R2201ET0266 | preflexion | | 1 | 1 | | | | | | 2 |
| R2201ET0267 | preflexion | | | 1 | | | 1 | | | 2 |
| R2201ET0278 | preflexion | | | | | | 2 | | | 2 |
| R2201ET0280 | preflexion | | 2 | | | | | | | 2 |
| R2201ET0286 | preflexion | | | | | | 3 | | | 3 |
| R2201ET0338 | preflexion | | | | | | 3 | | | 3 |
| R2201ET0339 | preflexion | | | | | | 2 | | | 2 |
| R2201ET0350 | preflexion | | | | 3 | | 3 | | | 6 |
| R2201ET0393 | preflexion | | | | | | 2 | | | 2 |
| R2201ET0401 | preflexion | | | 1 | | | 1 | | | 2 |
| R2201ET0410 | preflexion | | 1 | | | | | | | 1 |
| R2201ET0412 | preflexion | | | | | | 2 | | | 2 |
| R2201ET0435 | preflexion | | | | | | 2 | | | 2 |
| R2201ET1122 | preflexion | | 1 | | | 1 | 4 | | | 6 |
| R2201ET1211 | preflexion | | | 2 | | | 1 | | | 3 |
| R2201ET1683 | preflexion | | | | | | 2 | | | 2 |
| R2201ET1687 | preflexion | | 4 | | | | | | | 4 |
| R2201ET1690 | preflexion | | 1 | | | | 1 | | | 2 |
| R2201ET1693 | preflexion | | 1 | | | | | | | 1 |
| R2201FZ0349 | preflexion | | 1 | 1 | | | 1 | | | 3 |
| R2201FZ0350 | preflexion | | | 1 | | | 1 | | 2 | 4 |
| R2201FZ0351 | preflexion | | | | | 1 | 3 | | | 4 |
| R2201FZ0352 | preflexion | | | | | | 4 | | | 4 |
| R2201FZ0402 | preflexion | | | 1 | | | 2 | | | 3 |
| R2201FZ0404 | preflexion | | | | | | 3 | | | 3 |
| R2201FZ0407 | preflexion | | | | 2 | | 2 | | | 4 |
| R2201ET0081 | flexion | | 1 | | | | 1 | | | 2 |
| R2201ET0125 | flexion | 3 | | | | | 3 | | | 6 |
| R2201ET0210 | flexion | | | 3 | | | 1 | | | 4 |
| R2201ET0284 | flexion | | 3 | | | | | | | 3 |
| R2201ET0348 | flexion | | | | | | 3 | | | 3 |
| R2201ET0424 | flexion | | | | | 1 | 3 | | | 4 |
| R2201ET0428 | flexion | | | | | | 1 | | | 1 |
| R2201ET0678 | flexion | | | 1 | | | 5 | | | 6 |
| R2201ET0680 | flexion | | | | | | 7 | | | 7 |
| R2201ET0682 | flexion | | | 2 | | | | | | 2 |
| R2201ET0697 | flexion | | | | | | 5 | | | 5 |
| R2201ET0911 | flexion | | | | 1 | 1 | 2 | | | 4 |
| R2201ET1004 | flexion | | | 2 | | | 2 | | | 4 |
| R2201ET1005 | flexion | | | 1 | | | 1 | | | 2 |
| R2201ET1074 | flexion | | | | 3 | | 2 | | | 5 |
| R2201ET1075 | flexion | | 1 | 2 | | | 3 | | 1 | 7 |
| R2201ET1104 | flexion | | 2 | 2 | 3 | | | | | 7 |
| R2201ET1121 | flexion | 1 | | | | | 3 | | | 4 |
| R2201ET1123 | flexion | | | 1 | | | | | | 1 |
| R2201ET1203 | flexion | | | | | 1 | 4 | | | 5 |
| R2201ET1794 | flexion | | 1 | | 1 | | , | | 1 | 3 |
| R2201ET1883 | flexion | | | 3 | 1 | | 1 | | | 5 |
| R2201FZ0362 | flexion | | | 2 | 4 | | 1 | | | 7 |
| R2201FZ0405 | flexion | | | | | | 3 | | | 3 |
| R2201FZ0408 | flexion | | | | | | 4 | | | 4 |
| R2201FZ0410 | flexion | | | 1 | | | 2 | | | 3 |
| R2201ET0268 | postflexion | | | | 2 | | 5 | | | 7 |
| R2201ET0269 | postflexion | | | 2 | | | | 1 | | 3 |
| R2201ET0270 | postflexion | | | | | 1 | 3 | | 1 | 5 |
| R2201ET0271 | postflexion | | | | | 4 | | | | 4 |
| R2201ET0342 | postflexion | | | | 1 | | | 1 | | 2 |

**Table S2** (continued)

| ID number | Lifestage | Cil | Naup | Cal | Cory | Clad | App | Fish | Other | Total |
|---|---|---|---|---|---|---|---|---|---|---|
| R2201ET0344 | postflexion | | | | | 1 | 4 | | | 5 |
| R2201ET0363 | postflexion | | | | | | | 1 | | 1 |
| R2201ET0375 | postflexion | | | | | 5 | 1 | 2 | | 8 |
| R2201ET0658 | postflexion | | | | | | 2 | | | 2 |
| R2201ET0659 | postflexion | | | 2 | | | 3 | | | 5 |
| R2201ET0660 | postflexion | | | | | | 5 | | | 5 |
| R2201ET0661 | postflexion | | | 1 | | | 1 | | | 2 |
| R2201ET0676 | postflexion | | | | 1 | | 2 | | | 3 |
| R2201ET0677 | postflexion | | | | | | 3 | | | 3 |
| R2201ET0681 | postflexion | | | 1 | | | 3 | | | 4 |
| R2201ET0904 | postflexion | | 1 | 1 | | 1 | 1 | | | 4 |
| R2201ET0905 | postflexion | | | | | | 3 | | | 3 |
| R2201ET1003 | postflexion | | | 1 | | 2 | 2 | | | 5 |
| R2201ET1006 | postflexion | | | 2 | | 1 | 2 | | | 5 |
| R2201ET1058 | postflexion | | | 1 | | 2 | 1 | | 1 | 5 |
| R2201ET1073 | postflexion | | | | | 3 | 1 | | | 4 |
| R2201ET1084 | postflexion | | | | | | 7 | | | 7 |
| R2201ET1086 | postflexion | | | | | | | 1 | | 1 |
| R2201ET1105 | postflexion | | | 2 | | 1 | | | | 3 |
| R2201ET1881 | postflexion | | | | 2 | 2 | 1 | | | 5 |
| R2201ET1882 | postflexion | | | | | | | 1 | | 1 |
| R2201FZ0346 | postflexion | | | 1 | | | | | | 1 |
| R2201FZ0347 | postflexion | | | 1 | | | 1 | | 1 | 3 |
| R2201FZ0348 | postflexion | | 1 | 1 | | | 2 | | | 4 |
| R2201FZ0353 | postflexion | 1 | | 1 | | | 3 | | | 5 |
| R2201FZ0354 | postflexion | | 1 | | 1 | 1 | | | | 3 |
| R2201FZ0355 | postflexion | | | | | 1 | 6 | | | 7 |
| R2201FZ0356 | postflexion | | | 1 | | | 2 | | | 3 |
| R2201FZ0357 | postflexion | | 2 | | | | 1 | | | 3 |
| R2201FZ0358 | postflexion | | | 1 | | | 2 | | | 3 |
| R2201FZ0359 | postflexion | | | | | | | 2 | | 2 |
| R2201FZ0361 | postflexion | | | | 5 | 1 | 1 | | | 7 |
| R2201FZ0364 | postflexion | | | 1 | 1 | 2 | | | | 4 |
| R2201FZ0365 | postflexion | 3 | | 1 | 3 | 2 | 1 | | | 10 |
| R2201FZ0367 | postflexion | | | | | 2 | 4 | | | 6 |
| R2201FZ0369 | postflexion | | | | 1 | | 1 | | | 2 |
| R2201FZ0371 | postflexion | | | | | | 5 | | | 5 |
| R2201FZ0373 | postflexion | | | | | | 3 | | | 3 |
| R2201FZ0374 | postflexion | | | | | | 3 | | | 3 |
| R2201FZ0375 | postflexion | | | 1 | 1 | | 4 | | | 6 |
| R2201FZ0376 | postflexion | | | | 1 | | | | | 1 |
| R2201FZ0378 | postflexion | | 1 | | | 1 | 2 | | | 4 |
| R2201FZ0379 | postflexion | | | | | | 5 | | | 5 |
| R2201FZ0380 | postflexion | | | | | | 6 | | | 6 |
| R2201FZ0381 | postflexion | | | | | | | 1 | | 1 |
| R2201FZ0383 | postflexion | | | | | | 9 | | | 9 |
| R2201FZ0384 | postflexion | 3 | | | | | 11 | | | 14 |
| R2201FZ0385 | postflexion | | | 2 | 1 | | 3 | | 1 | 7 |
| R2201FZ0386 | postflexion | | | | | | 6 | | | 6 |
| R2201FZ0387 | postflexion | | | | | | 4 | | | 4 |
| R2201FZ0388 | postflexion | | | | | | | 1 | | 1 |
| R2201FZ0390 | postflexion | | | 1 | | 1 | 4 | | | 6 |
| R2201FZ0391 | postflexion | | | | | | 4 | | | 4 |
| R2201FZ0392 | postflexion | | | | | 1 | 2 | | | 3 |
| R2201FZ0393 | postflexion | | | | | 1 | 6 | | | 7 |
| R2201FZ0394 | postflexion | | | 1 | | 1 | 4 | | | 6 |
| R2201FZ0395 | postflexion | | | | | | 5 | | | 5 |
| R2201FZ0396 | postflexion | | | 1 | | | 1 | | | 2 |
| R2201FZ0397 | postflexion | | | | | | 5 | | | 5 |
| R2201FZ0398 | postflexion | | | | | 2 | 3 | | | 5 |
| R2201FZ0399 | postflexion | | | | | | 2 | | | 2 |
| R2201FZ0400 | postflexion | | | | | | 4 | | | 4 |
| R2201FZ0401 | postflexion | | | | | | 3 | | | 3 |
| R2201FZ0403 | postflexion | | 1 | | | | 5 | | | 6 |
| R2201FZ0406 | postflexion | | | 1 | | | | | | 1 |
| R2201FZ0409 | postflexion | | | | | | 7 | | | 7 |
| | Numbers | 15 | 49 | 58 | 39 | 46 | 308 | 11 | 9 | 535 |
| | % | 2.8 | 9.2 | 10.8 | 7.3 | 8.6 | 57.6 | 2.1 | 1.7 | |
| | Preflexion | 4 | 34 | 10 | 6 | 4 | 61 | 0 | 3 | 122 |
| | Flexion | 4 | 8 | 20 | 13 | 3 | 57 | 0 | 2 | 107 |
| | Postflexion | 7 | 7 | 28 | 20 | 39 | 190 | 11 | 4 | 306 |
| | % Preflexion | 3.3 | 27.9 | 8.2 | 4.9 | 3.3 | 50.0 | 0.0 | 2.5 | |
| | % Flexion | 3.7 | 7.5 | 18.7 | 12.1 | 2.8 | 53.3 | 0.0 | 1.9 | |
| | % Postflexion | 2.3 | 2.3 | 9.2 | 6.5 | 12.7 | 62.1 | 3.6 | 1.3 | |

**Table S3.** Sizes and percent abundances of appendicularian species from noontime net tows in the upper 30 m duringexperimental cycles C1-C4.

**SUMMARY: Species Mean Trunk and Tail Lengths (mm)**

| Species | Cycle 1 | | Cycle 2 | | Cycle 3 | | Cycle 4 | |
|---|---|---|---|---|---|---|---|---|
| | Trunk | Tail | Trunk | Tail | Trunk | Tail | Trunk | Tail |
| *Oikopleura parva* | 0.58 | 2.03 | 0.62 | 2.02 | 0.66 | 2.09 | 0.63 | 2.34 |
| *Oikopleura rufrescens* | 0.51 | 2.55 | 0.51 | 2.07 | 0.57 | 2.49 | 0.61 | 3.32 |
| *Oikopleura longicauda* | 0.37 | 2.26 | 0.34 | 2.08 | 0.36 | 2.21 | 0.48 | 2.63 |
| *Oikopleura spp.* | 0.26 | 2.07 | 0.33 | 2.09 | 0.33 | 2.03 | 0.32 | 2.04 |
| *Megalocercus huxleyi* | | | 1.65 | 6.50 | | | 0.60 | 2.40 |
| *Fritillaria borealis sargassi* | 1.08 | 1.48 | 4.50 | 5.00 | 1.02 | 1.70 | 1.04 | 1.46 |
| *Fritillaria haplostoma* | 1.33 | 1.37 | 1.00 | 1.05 | 1.43 | 1.80 | 1.01 | 1.31 |
| *Fritillaria megachile* | 1.75 | 1.75 | | | | | 1.04 | 2.00 |

**SUMMARY: Species Percent Abundances (%)**

| Species | Cycle 1 | Cycle 2 | Cycle 3 | Cycle 4 |
|---|---|---|---|---|
| *Oikopleura parva* | 12 | 22 | 10 | 7 |
| *Oikopleura rufrescens* | 2 | 5 | 16 | 7 |
| *Oikopleura longicauda* | 10 | 35 | 35 | 44 |
| *Oikopleura spp.* | 63 | 29 | 24 | 26 |
| *Megalocercus huxleyi* | 0 | 4 | 0 | 1 |
| *Fritillaria borealis sargassi* | 7 | 2 | 11 | 5 |
| *Fritillaria haplostoma* | 4 | 4 | 3 | 7 |
| *Fritillaria megachile* | 2 | 0 | 0 | 3 |

**DETAILED DATA: Individual specimens analyzed from experimental cycles**

| Species | Cycle 1 | | Cycle 2 | | Cycle 3 | | Cycle 4 | |
|---|---|---|---|---|---|---|---|---|
| | Trunk | Tail | Trunk | Tail | Trunk | Tail | Trunk | Tail |
| *Oikopleura parva* | 0.67 | 2.1 | 0.60 | 2.0 | 0.60 | 1.9 | 0.60 | 2.5 |
| | 0.62 | 2.4 | 0.60 | 2.0 | 0.60 | 2.3 | 0.43 | 2.0 |
| | 0.55 | 1.7 | 0.55 | 2.0 | 0.90 | 2.7 | 0.53 | 2.1 |
| | 0.70 | 1.9 | 0.55 | 1.9 | 0.60 | 2.0 | 0.80 | 2.5 |
| | 0.50 | 1.6 | 0.50 | 1.7 | 0.90 | 2.4 | 0.71 | 2.1 |
| | 0.50 | 2.2 | 0.45 | 1.9 | 0.52 | 1.6 | 0.70 | 1.9 |
| | 0.65 | 2.2 | 0.70 | 2.2 | 0.62 | 1.7 | 0.70 | 2.1 |
| | 0.55 | 2.0 | 0.70 | 2.2 | 0.61 | 2.0 | 0.45 | 2.1 |
| | 0.70 | 2.4 | 0.80 | 2.4 | 0.62 | 2.2 | 0.65 | 3.1 |
| | 0.40 | 1.8 | 0.70 | 1.9 | | | 0.75 | 2.4 |
| | | | 0.64 | 2.0 | | | 0.60 | 2.9 |
| | | | 0.70 | 2.0 | | | | |

| Table S3 (continued) | Cycle 1 | | Cycle 2 | | Cycle 3 | | Cycle 4 | |
|---|---|---|---|---|---|---|---|---|
| Species | Trunk | Tail | Trunk | Tail | Trunk | Tail | Trunk | Tail |
| *Oikopleura rufrescens* | 0.51 | 2.7 | 0.50 | 2.0 | 0.50 | 2.0 | 0.85 | 4.0 |
| | 0.50 | 2.4 | 0.42 | 1.6 | 0.50 | 2.4 | 0.65 | 3.6 |
| | | | 0.60 | 2.6 | 0.64 | 2.4 | 0.45 | 2.8 |
| | | | | | 0.50 | 2.4 | 0.40 | 3.6 |
| | | | | | 0.64 | 2.4 | 0.60 | 3.6 |
| | | | | | 0.64 | 2.4 | 0.75 | 3.3 |
| | | | | | 0.58 | 2.5 | 0.60 | 2.8 |
| | | | | | 0.52 | 2.3 | 0.65 | 3.0 |
| | | | | | 0.64 | 3.1 | 0.82 | 4.7 |
| | | | | | 0.40 | 2.0 | 0.60 | 2.2 |
| | | | | | 0.70 | 3.3 | 0.35 | 3.0 |
| | | | | | 0.65 | 3.0 | | |
| | | | | | 0.50 | 2.4 | | |
| | | | | | 0.52 | 2.2 | | |
| *Oikopleura longicauda* | 0.34 | 2.2 | 0.39 | 2.2 | 0.48 | 2.6 | 0.38 | 2.1 |
| | 0.30 | 1.7 | 0.28 | 2.7 | 0.32 | 1.8 | 0.45 | 2.2 |
| | 0.30 | 1.8 | 0.46 | 2.2 | 0.28 | 1.8 | 0.40 | 2.4 |
| | 0.52 | 3.4 | 0.20 | 0.9 | 0.54 | 2.6 | 0.35 | 2.5 |
| | 0.38 | 1.8 | 0.36 | 2.0 | 0.26 | 1.8 | 0.44 | 2.6 |
| | 0.36 | 2.4 | 0.30 | 1.9 | 0.38 | 2.8 | 0.19 | 2.2 |
| | 0.40 | 2.4 | 0.40 | 2.4 | 0.38 | 1.8 | 0.60 | 3.2 |
| | 0.36 | 2.4 | 0.40 | 2.0 | 0.32 | 2.1 | 1.00 | 4.0 |
| | | | 0.14 | 1.2 | 0.44 | 2.6 | 0.67 | 2.4 |
| | | | 0.30 | 1.4 | 0.30 | 2.6 | 0.65 | 3.0 |
| | | | 0.23 | 2.2 | 0.44 | 2.8 | 0.50 | 2.5 |
| | | | 0.40 | 2.4 | 0.40 | 2.6 | 0.50 | 3.0 |
| | | | 0.26 | 1.3 | 0.38 | 1.8 | 0.55 | 2.0 |
| | | | 0.41 | 1.8 | 0.46 | 2.0 | 0.45 | 2.6 |
| | | | 0.45 | 2.4 | 0.32 | 2.0 | 0.50 | 2.7 |
| | | | 0.31 | 2.4 | 0.50 | 2.4 | 0.50 | 2.6 |
| | | | 0.36 | 2.8 | 0.36 | 2.1 | 0.45 | 2.6 |
| | | | 0.38 | 2.8 | 0.36 | 2.2 | 0.51 | 2.8 |
| | | | 0.38 | 2.6 | 0.40 | 2.4 | 0.50 | 2.4 |
| | | | | | 0.33 | 2.6 | 0.40 | 3.0 |
| | | | | | 0.30 | 2.6 | 0.50 | 2.4 |
| | | | | | 0.27 | 1.4 | 0.50 | 2.6 |
| | | | | | 0.24 | 1.6 | 0.55 | 3.1 |
| | | | | | 0.16 | 1.4 | 0.55 | 2.4 |
| | | | | | 0.35 | 2.4 | 0.62 | 2.7 |

| Table S3 (continued) | Cycle 1 | | Cycle 2 | | Cycle 3 | | Cycle 4 | |
|---|---|---|---|---|---|---|---|---|
| Species | Trunk | Tail | Trunk | Tail | Trunk | Tail | Trunk | Tail |
| *Oikopleura longicauda* | | | | | 0.40 | 2.6 | 0.57 | 3.0 |
| (continued) | | | | | 0.32 | 2.0 | 0.55 | 2.6 |
| | | | | | 0.35 | 2.6 | 0.50 | 3.4 |
| | | | | | 0.30 | 2.2 | 0.40 | 1.9 |
| | | | | | 0.28 | 1.8 | 0.45 | 2.6 |
| | | | | | 0.40 | 2.4 | 0.80 | 3.4 |
| | | | | | | | 0.65 | 3.2 |
| | | | | | | | 0.40 | 2.4 |
| | | | | | | | 0.55 | 2.4 |
| | | | | | | | 0.40 | 2.3 |
| | | | | | | | 0.60 | 3.4 |
| | | | | | | | 0.60 | 3.4 |
| | | | | | | | 0.45 | 2.3 |
| | | | | | | | 0.40 | 2.5 |
| | | | | | | | 0.50 | 2.2 |
| | | | | | | | 0.50 | 2.7 |
| | | | | | | | 0.53 | 3.0 |
| | | | | | | | 0.60 | 3.0 |
| | | | | | | | 0.55 | 3.6 |
| | | | | | | | 0.45 | 2.4 |
| | | | | | | | 0.50 | 2.7 |
| | | | | | | | 0.45 | 2.7 |
| | | | | | | | 0.35 | 3.8 |
| | | | | | | | 0.42 | 2.8 |
| | | | | | | | 0.45 | 2.8 |
| | | | | | | | 0.32 | 2.4 |
| | | | | | | | 0.30 | 2.2 |
| | | | | | | | 0.35 | 2.4 |
| | | | | | | | 0.36 | 2.5 |
| | | | | | | | 0.35 | 2.5 |
| | | | | | | | 0.30 | 1.9 |
| | | | | | | | 0.17 | 1.2 |
| | | | | | | | 0.40 | 1.9 |
| | | | | | | | 0.40 | 2.8 |
| | | | | | | | 0.40 | 2.4 |
| | | | | | | | 0.50 | 2.6 |
| | | | | | | | 0.45 | 2.3 |
| | | | | | | | 0.40 | 2.0 |
| | | | | | | | 0.52 | 2.4 |
| | | | | | | | 0.80 | 3.4 |
| | | | | | | | 0.40 | 2.5 |

| Table S3 (continued) | Cycle 1 | | Cycle 2 | | Cycle 3 | | Cycle 4 | |
|---|---|---|---|---|---|---|---|---|
| **Species** | **Trunk** | **Tail** | **Trunk** | **Tail** | **Trunk** | **Tail** | **Trunk** | **Tail** |
| *Oikopleura* spp. | 0.30 | 2.1 | 0.20 | 2.0 | 0.28 | 2.6 | 0.30 | 1.6 |
| | 0.22 | 1.7 | 0.40 | 2.3 | 0.38 | 2.2 | 0.30 | 2.3 |
| | 0.20 | 2.0 | 0.30 | 2.4 | 0.36 | 2.0 | 0.24 | 1.8 |
| | 0.24 | 1.6 | 0.36 | 2.4 | 0.26 | 1.6 | 0.40 | 2.1 |
| | 0.22 | 1.8 | 0.36 | 2.6 | 0.40 | 1.4 | 0.30 | 2.5 |
| | 0.31 | 2.6 | 0.35 | 2.4 | 0.32 | 2.0 | 0.70 | 2.9 |
| | 0.22 | 2.2 | 0.45 | 1.2 | 0.44 | 2.5 | 0.35 | 3.3 |
| | 0.18 | 1.2 | 0.38 | 1.9 | 0.24 | 1.5 | 0.35 | 2.5 |
| | 0.24 | 1.2 | 0.20 | 3.2 | 0.60 | 2.3 | 0.30 | 2.4 |
| | 0.24 | 1.8 | 0.28 | 1.4 | 0.30 | 1.8 | 0.24 | 2.3 |
| | 0.22 | 2.1 | 0.31 | 1.3 | 0.14 | 1.3 | 0.35 | 3.3 |
| | 0.36 | 2.6 | 0.25 | 2.0 | 0.30 | 2.4 | 0.50 | 3.0 |
| | 0.10 | 1.7 | 0.45 | 2.2 | 0.46 | 2.8 | 0.30 | 2.2 |
| | 0.10 | 1.8 | | 1.8 | 0.36 | 2.4 | 0.29 | 2.0 |
| | 0.30 | 2.2 | | 1.8 | 0.12 | 0.9 | 0.34 | 2.6 |
| | 0.30 | 2.4 | | 2.6 | 0.17 | 1.4 | 0.34 | 0.2 |
| | 0.28 | 1.6 | | | 0.30 | 1.5 | 0.25 | 0.2 |
| | 0.30 | 1.6 | | | 0.35 | 3.0 | 0.45 | 0.3 |
| | 0.28 | 2.2 | | | 0.45 | 2.4 | 0.20 | 1.7 |
| | 0.24 | 2.1 | | | | 2.4 | 0.40 | 2.7 |
| | 0.28 | 1.8 | | | | 2.2 | 0.35 | 2.8 |
| | 0.24 | 2.4 | | | | | 0.33 | 2.2 |
| | 0.24 | 2.6 | | | | | 0.30 | 1.5 |
| | 0.38 | 2.8 | | | | | 0.28 | 2.3 |
| | 0.30 | 2.6 | | | | | 0.21 | 1.9 |
| | 0.34 | 2.4 | | | | | 0.25 | 1.6 |
| | 0.30 | 1.8 | | | | | 0.24 | 1.2 |
| | 0.20 | 2.0 | | | | | 0.06 | 0.2 |
| | 0.26 | 2.0 | | | | | 0.40 | 2.7 |
| | 0.24 | 2.4 | | | | | 0.35 | 2.6 |
| | 0.28 | 2.2 | | | | | 0.22 | 2.0 |
| | 0.36 | 0.6 | | | | | 0.40 | 2.7 |
| | 0.24 | 1.6 | | | | | 0.20 | 1.3 |
| | 0.24 | 2.0 | | | | | | 1.7 |
| | 0.24 | 1.8 | | | | | | 1.1 |
| | 0.30 | 2.3 | | | | | | 2.9 |
| | 0.40 | 2.7 | | | | | | 1.7 |
| | 0.24 | 1.6 | | | | | | 1.9 |
| | 0.20 | 1.8 | | | | | | 3.3 |

| **Table S3** (continued) | **Cycle 1** | | **Cycle 2** | | **Cycle 3** | | **Cycle 4** | |
|---|---|---|---|---|---|---|---|---|
| **Species** | **Trunk** | **Tail** | **Trunk** | **Tail** | **Trunk** | **Tail** | **Trunk** | **Tail** |
| *Oikopleura* spp. | 0.24 | 1.3 | | | | | | |
| (continued) | 0.34 | 2.4 | | | | | | |
| | 0.24 | 2.2 | | | | | | |
| | 0.20 | 2.0 | | | | | | |
| | 0.18 | 2.4 | | | | | | |
| | 0.30 | 3.4 | | | | | | |
| | 0.28 | 2.7 | | | | | | |
| | 0.30 | 2.2 | | | | | | |
| | 0.32 | 2.3 | | | | | | |
| | 0.26 | 1.9 | | | | | | |
| | | 2.5 | | | | | | |
| | | 2.2 | | | | | | |
| | | 2.4 | | | | | | |
| *Megalocercus huxleyi* | | | 1.90 | 7.2 | | | 0.60 | 2.4 |
| | | | 1.40 | 5.8 | | | | |
| *Fritillaria borealis sargassi* | 1.00 | 1.4 | 4.50 | 5.0 | 1.00 | 2.0 | 1.10 | 1.5 |
| | 1.10 | 1.7 | | | 1.00 | 1.6 | 1.20 | 1.9 |
| | 1.20 | 1.6 | | | 1.00 | 1.9 | 1.20 | 1.8 |
| | 1.10 | 1.5 | | | 1.20 | 2.4 | 0.90 | 1.3 |
| | 1.00 | 1.2 | | | 1.20 | 1.6 | 1.00 | 1.3 |
| | | 1.5 | | | 1.20 | 1.8 | 1.00 | 1.2 |
| | | | | | 1.10 | 2.0 | 0.90 | 1.2 |
| | | | | | 0.80 | 1.3 | | |
| | | | | | 0.88 | 1.2 | | |
| | | | | | 0.85 | 1.2 | | |
| *Fritillaria haplostoma* | 1.30 | 1.3 | 1.20 | 1.2 | 1.40 | 1.8 | 0.80 | 0.9 |
| | 1.30 | 1.8 | 0.80 | 0.9 | 1.60 | 1.8 | 1.40 | 1.6 |
| | 1.40 | 1.0 | | | 1.30 | 1.8 | 1.40 | 2.2 |
| | | | | | | | 0.55 | 0.8 |
| | | | | | | | 0.70 | 1.1 |
| | | | | | | | 0.84 | 0.9 |
| | | | | | | | 0.55 | 0.8 |
| | | | | | | | 1.20 | 1.5 |
| | | | | | | | 1.20 | 1.5 |
| | | | | | | | 1.50 | 1.8 |
| *Fritillaria megachile* | 1.40 | 1.2 | | | | | 0.85 | 2.9 |
| | 2.10 | 2.3 | | | | | 1.20 | 1.9 |
| | | | | | | | 1.04 | 2.2 |
| | | | | | | | 1.00 | 1.6 |
| | | | | | | | 1.10 | 1.4 |
